# Whole-Exome Sequencing Identifies Damaging *de novo* Variants in Anencephalic Cases

**DOI:** 10.3389/fnins.2019.01285

**Published:** 2019-11-29

**Authors:** Linlin Wang, Aiguo Ren, Tian Tian, Nan Li, Xuanye Cao, Peng Zhang, Lei Jin, Zhiwen Li, Yan Shen, Bo Zhang, Richard H. Finnell, Yunping Lei

**Affiliations:** ^1^Institute of Reproductive and Child Health, National Health Commission Health Key Laboratory of Reproductive Health, Department of Epidemiology and Biostatistics, School of Public Health, Peking University Health Science Center, Beijing, China; ^2^Center for Precision Environmental Health, Department of Molecular and Cellular Biology, Baylor College of Medicine, Houston, TX, United States; ^3^Institute of Biophysics, Chinese Academy of Sciences, Beijing, China; ^4^Key Laboratory of Cell Proliferation and Differentiation of the Ministry of Education, College of Life Sciences, Peking University, Beijing, China

**Keywords:** neural tube defects, *de novo* variants, whole-exome sequencing, WIPI1, anencephaly

## Abstract

**Background:**

Anencephaly is a lethal neural tube defect (NTD). Although variants in several genes have been implicated in the development of anencephaly, a more complete picture of variants in the genome, especially *de novo* variants (DNVs), remains unresolved. We aim to identify DNVs that play an important role in the development of anencephaly by performing whole-exome DNA sequencing (WES) of proband–parent trios.

**Results:**

A total of 13 DNVs were identified in 8 anencephaly trios by WES, including two loss of function (LoF) variants detected in pLI > 0.9 genes (*SPHKAP*, c.2629_2633del, and *NCOR1*, p.Y1907X). Damaging DNVs were identified in 61.5% (8/13) of the anencephalic cases. Independent validation was conducted in an additional 502 NTD cases. Gene inactivation using targeted morpholino antisense oligomers and rescue assays were conducted in zebrafish, and transfection expression in HEK293T cells. Four DNVs in four cases were identified and predicted to alter protein function, including p.R328Q in WD repeat domain phosphoinositide-interacting 1 (WIPI1). Three variants, p.G313R, p.T418M, and p.L406P, in the WIPI1 gene were identified from the independent replication cohort consisting of 502 cases. Functional analysis suggested that the *wipi1* p.L406P and p.R328Q variants most likely displayed loss-of-function effects during embryonic development.

**Conclusion:**

*De novo* damaging variants are the main culprit for majority of anencephalic cases. Missense variants in WIPI1 may play a role in the genetic etiology of anencephaly, and LoF variants in *SPHKAP* and *NCOR1* may also contribute to anencephaly. These findings add to our existing understanding of the genetic mechanisms of NTD formation.

## Introduction

Neural tube defects (NTDs) are severe congenital malformations of the central nervous system (including anencephaly, spina bifida, and encephalocele) that are caused by a partial or incomplete closure of the neural tube during embryogenesis (Wallingford et al., 2013). Infants with anencephaly are mostly stillborn or die shortly after birth, while infants with spina bifida and encephalocele may survive but suffer from physical and developmental disabilities with varying degrees of severity. NTDs in humans are considered to have a multifactorial etiology, with contributions from both genetic and environmental factors (Kibar et al., 2007a; Haddow, 2011; Wallingford et al., 2013; Kang et al., 2018). Despite a long history of etiological studies in humans (Blom et al., 2006; Wallingford et al., 2013; Murdoch et al., 2014; Wilde et al., 2014), the causative mechanism underlying the development of NTDs remains largely unknown.

Etiologically, NTDs are understood to have a significant genetic component with an estimated heritability of 60% (Bassuk and Kibar, 2009). While over ∼300 causative genes have been linked to mouse NTDs (Harris and Juriloff, 2007, 2010; Juriloff and Harris, 2012; Wilde et al., 2014), identification of NTD genes in humans is difficult, and the predisposing genetic factors for human NTDs are still unclear. To characterize these genetic underpinnings, efforts have been made toward identifying common, rare, or *de novo* DNA variants that contribute to the occurrence of NTDs. Very few common DNA variations, such as variations in folate-related genes including methylene-tetrahydrofolate reductase (MTHFR), have been identified using genetic association studies; and these common variations might at most confer a modest risk and account for only a very small portion of disease heritability. The other “undiscovered” heritability may be attributed to rare variants that are uncommon in the general population but probably producing larger adverse genetic effects on NTDs than common variants (Manolio et al., 2009). As described by Chen et al. (2018), the genetic contribution in the form of multiple singleton loss of function (LoF) variants can come from any number of KEGG ontogeny groups spread over the genome.

Rare variant discovery has previously proceeded on sequencing of candidate genes identified using *a priori* data from animal models or based on functional relevance. According to research clues provided by animal models, the strategy of targeting candidate genes has successfully identified a number of causative variations in genes primarily from the planar cell polarity (PCP) signaling pathway that controls the process by which cells become polarized within the plane of an epithelium in numerous tissues in both *Drosophila* and in vertebrates (Simons and Mlodzik, 2008; Gray et al., 2011). For example, rare variants in the core PCP genes *CELSR1*, *FZD6*, *PRICKLE1*, *DVL2*, *VANGL1*, and *VANGL2* (Kibar et al., 2007b; Lei et al., 2010; Bosoi et al., 2011; Seo et al., 2011; Allache et al., 2012; De Marco et al., 2012, 2013; Juriloff and Harris, 2012) have been established as human NTD risk factors. However, candidate gene studies in NTDs are slow and inherently biased, and have faced limited success in identifying more causative genes predisposing to human NTDs, further demonstrating the need for novel alternative approaches.

With rapid development of high-throughput-sequencing technology and the concurrent reduction in sequencing costs, whole-exome sequencing (WES) now is becoming a powerful approach to investigating new candidate genes and new variants associated with human disease (Riviere et al., 2012; Nava et al., 2014; Schwarze et al., 2018). In particular, the application of WES in parent–offspring trios or multiplex families has been successful in identifying candidate pathogenic *de novo* variants (DNVs) in patients with neurodevelopmental disorders (Yates et al., 2017), such as intellectual disability (Rauch et al., 2012), autism spectrum disorder (Neale et al., 2012), and schizophrenia (Girard et al., 2011). To date, only one Canadian research group has published WES data from 43 trios affected with NTDs (35 myelomeningocele and 8 anencephaly), and another eight families with both forms of open and closed NTDs (including seven myelomeningocele, four spina bifida occulta, one anencephaly, and so on), demonstrating an important role that LoF DNVs play in the development of NTDs (Lemay et al., 2015, 2019).

In the present study, we focus on the detection of DNVs from cases with the most severe subtype of NTDs, anencephaly. We conducted WES in 13 parent–offspring trios of a child with anencephaly to call variants in each sample of a trio independently, and then identify putative DNVs by comparing offspring against parental genotypes with Mendelian inconsistency. Thus, we identified DNVs expected to affect neurodevelopment and contribute to the occurrence of anencephaly. We subsequently sequenced the gene that carries the DNVs in a larger independent NTD cohort. Knock down experiments using targeted morpholino (MO) antisense oligomers and rescue assays in zebrafish as well as transfection expression studies in HEK293T cells were performed to explore the functional relevance of candidate variants.

## Materials and Methods

### Study Subjects

Subjects were recruited from five rural counties (Taigu, Pingding, Xiyang, Shouyang, and Zezhou) in Shanxi Province of northern China from 2011 to 2014. Cases with a confirmed prenatal diagnosis of an NTD were ascertained through a population-based birth defect surveillance program (Liu et al., 2016). Skin tissues taken from infants were collected at delivery or at the time of termination of NTD affected pregnancies by experienced pathologists, and kept frozen at −80°C until used for various analyses. Blood samples from parents were collected at same time and stored at −80°C until needed. The study protocol was approved by the institutional review board of Peking University. Written informed consent was obtained from all mothers before the study.

### Whole-Exome Sequencing and Variant Prediction

We performed WES on 13 trios comprised of a child with a cranial NTD (2 anencephaly only; 11 anencephaly and spina bifida) and unaffected parents to identify DNVs in their exomes. Briefly, exomes were captured from genomic DNA using Agilent SureSelect Human All Exon Capture V4 kit (Agilent Technologies, Mississauga, ON, Canada) and then sequenced on an Illumina HiSeq 2000 sequencer using 101-bp paired-end reads. Supplementary Materials detail the methods used for the whole-exome data analysis. The summary of sequencing QA/QC is listed in Supplementary Table S1. We identified DNVs by excluding all variants found in the proband that were transmitted by one or both of the parents. We manually checked the sequencing context for all coding variants via Integrative Genomics Viewer (IGV) (Thorvaldsdottir et al., 2013). The sensitivity and specificity of our pipeline was tested with the genome in a bottle^1^ (Zook et al., 2016).

PolyPhen^2^, SIFT^3^, and PROVEAN prediction^4^ algorithms were employed to predict the impact of exome coding variants on protein function. Clustal-Omega 1.2.1 software was used to estimate the conservation of amino acids that were changed by a variant. Residues were considered to be highly conserved if there was no variation in amino acid properties observed across the compared six orthologous proteins, including five mammalian orthologous proteins plus zebrafish.

### WIPI1 Sanger DNA Resequencing and Case–Control Burden Test

Potential DNVs identified by exome sequencing were validated by Sanger sequencing in the child and both parents. Primer5.0 was used to design the specific primers used for the PCR amplification and direct sequencing of the surrounding region of each variant. Sequencing reactions were performed on G-50-purified PCR products with the BigDye Terminator kit. Sequencing products were run on an ABI Prism 3730 DNA Analyzer (Applied Biosystems). Sequences were analyzed using Mutation Surveyor software v3.97 (SoftGenetics) and were verified by manual inspection. To perform WIPI1 case–control burden test, we extracted East Asian rare (MAF < 0.001) predicted-to-be-damaging variants from gnomAD database^5^. Fisher’s exact test was used for case–control burden test.

### Parental Testing

Parental testing was carried out with the AmpFl STR SGM plus kit (Applied Biosystems) to exclude false paternity, and DNA inversion to determine whether a variant had occurred *de novo*.

### Multiplex PCR Amplification and Next-Generation Sequencing

Multiplex PCR amplification and next-generation sequencing were performed to screen for DNA variants along the entire coding regions and intron–exon boundaries of the targeted *WIPI1* gene. Detailed methods of PCR amplification and sequencing are provided in the Supplementary Materials.

### Zebrafish Morpholino and mRNA Injections

Wild-type Tübingen zebrafish strains were raised under standard conditions at 28.5°C. Injections were performed in the fertilized egg at its one cell stage. MO injection was performed with a splice *wipi1*-MO (5′-CTGTGTTGTGTTTACCTGTCTGTTG-3′). MOs were designed by Gene Tools, LLC (Philomath, OR, United States). To produce wipi1 mRNA, the full coding *wipi1* transcript (NM_017983) was PCR amplified and cloned into a pCMV6-Entry vector (Origene Technologies, MD, United States). The cDNA was transfected into *Escherichia coli* and the plasmid containing the *wipi1* cDNA was extracted and enzyme digested. Finally, the capped and polyadenylated *wipi1* mRNA was produced by *in vitro* transcription using mMESSAGE mMACHINETM T7 Transcription Kit (Ambion, Austin, TX, United States). The synthesized mRNA was diluted in phenol red to different concentrations for micro-injection. All injected embryos were observed at 24 and 48 h post-fertilization (hpf) and images of each phenotype were captured by using the CCD image system under bright field. To knockdown the translation of the zebrafish *wipi1*, different doses of MO reagent including 1, 2, 4, and 6 ng were injected. For the overexpression experiment, 100, 200, and 300 pg human *WIPI1* mRNA was injected into different embryos. In the rescue study, 100, 200, and 300 pg of human *WIPI1* WT, or mutant plasmids mRNA, were co-injected with 4 ng of the *wipi1-*MO. All embryos were independently assessed by two individuals to avoid bias and divided into four grades according to the shortening and bending of the body axis. The distribution of malformation of each group was compared by Pearson’s χ^2^ test and Fisher’s exact test.

### Plasmids

Human *WIPI1* full-length cDNA was cloned into pIRES2-dsRed plasmid (pIRES2-dsRed-WIPI1). *WIPI1* missense changes R328Q, G313R, T418M, or L406P were introduced into pIRES2-dsRed-WIPI1 by QuikChange II Site-Directed Mutagenesis Kits (Agilent Technologies, Inc., CA, United States). All plasmids were validated by sequencing analyses.

### Cell Culture and Transfection

HEK293T cells were purchased from the Cell Bank of Chinese Academy of Sciences (Shanghai, China) and cultured according to the manufacturer’s protocols. HEK293T cells were plated into six-well plates and transfected with dsRed-tagged WIPI1 wild-type, R328Q, G313R, T418M, or L406P variant using Lipofectamine 2000 Transfection Reagent (Invitrogen) according to the manufacturer’s manual. Cells were plated at a density of 2.0 × 10^6^ cells per 100-mm Petri dish for real-time PCR, protein blotting, and subcellular localization analyses. Cells were incubated for 24 h at 37°C under an atmosphere containing 5% CO_2_ prior to use.

### Real-Time PCR

RNA was isolated from cells using Trizol (Invitrogen). DNase I digestion (DNA-free, Ambion) was used to remove genomic DNA; after that RNA was reverse-transcribed using random hexamers (Superscript VILO cDNA synthesis kit). The abundance of *WIPI1* mRNA was analyzed using real-time PCR reagent kit (PrimeScript^TM^ RT reagent Kit with gDNA Eraser and TB Green^TM^ Premix Ex Taq^TM^ II, TAKARA) on a Real-Time PCR system (Biorad, CFX96). Each sample was analyzed in triplicate. Relative quantification of each gene expression level was normalized according to the glyceraldehyde-3-phosphate dehydrogenase (GAPDH) expression.

### Western Blot

Protein was extracted on ice from cells using radioimmunoprecipitation assay (RIPA) buffer. Protein concentrations were determined using the Bradford assay. Western blotting was performed by conventional methods, with 50 μg of protein run per sample on NuPAGE 4–12% Bis–Tris gel (Life Technologies, Carlsbad, CA, United States), and transferred to a polyvinylidene difluoride (PVDF) membrane (XCell II Blot Module, Invitrogen). Primary antibodies were WIPI1 antibody (1:1000, Cell Signaling Technology, MA, United States) and Rabbit anti-GAPDH antibody (1:1000, Cell Signaling Technology, MA, United States). After incubation with secondary antibody anti-rabbit IgG (1:10,000, Cell Signaling Technology, MA, United States), blots were developed using ECL Prime (Solarbio, Beijing, China). ImageJ was used to detect densitometry data. Results were normalized with the GAPDH loading control. Independent experiments were carried out three times for each sample.

### Subcellular Localization

HEK293T cells were plated into six-well plates and transfected with dsRed-tagged *WIPI1* wild-type, R328Q, G313R, T418M, or L406P variant. Forty-eight hours later, the cells were washed three times with phosphate-buffered saline (PBS) and incubated 5 min with Hochest 3342 (1 μg/ml) (Invitrogen) and CellMask^TM^ Plasma Membrane Stains (Life Technologies, Carlsbad, CA, United States) (2 μg/ml) solution. The cells were subsequently washed in PBS three times and fixed in 4% paraformaldehyde (PFA in PBS) for 10 min at room temperature, followed by three more PBS washes. Cells were examined and photographed by a fluorescence microscopy (Olympus, BX51). The assay was repeated independently in three times.

### Statistical Analysis

Differences in proportions of zebrafish embryos with convergent extension defects between groups were examined with Pearson’s χ^2^ test or Fisher’s exact test. Independent samples *t*-test was used to identify the difference in expression of mRNA or protein between groups. Statistical analyses were conducted using SPSS 20.0. A two-tailed *P-*value of <0.05 was considered statistical significance.

## Results

### *De novo* Variants Identified in NTD-Affected Trios

**FIGURE 1:**
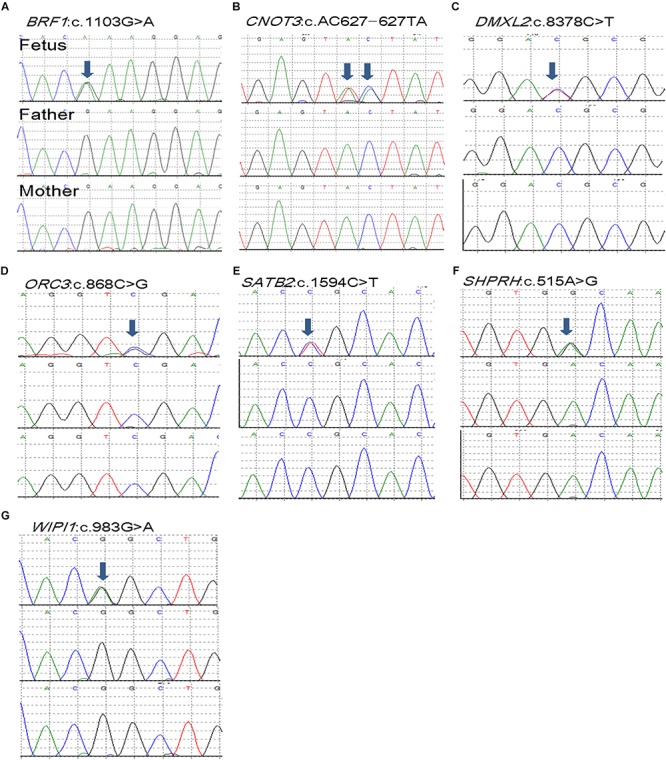
*De novo* mutations (DNMs) validation of selected seven DNVs in trios affected with anencephaly. Chromatograms of the three individuals from one trio (from top to bottom: fetus, father, and mother). **(A)** BRF1 *de novo* variant. **(B)** CNOT3 *de novo* variant. **(C)** DMXL2 *de novo* variant. **(D)** ORC3 *de novo* variant. **(E)** SATB2 *de novo* variant. **(F)** SHPRH *de novo* variant. **(G)** WIPI1 *de novo* variant.

A total of 13 coding DNVs in eight cases were identified. Seven of them were selected for validation by Sanger sequencing and all of them were confirmed as true positive (Table 1 and Figure 1). All bam file scripts were attached in Supplementary Figure S1. Two LoF variants were identified in *SPHKAP* (c.2629_2633del) and *NCOR1* (p.Y1907X) gene, respectively. One case carried three DNVs. Among the 13 DNVs, 9 of them were non-synonymous SNVs which were all predicted to be damaging by either SIFT or PolyPhen2. For all the eight non-synonymous SNVs carriers, at least one predicted to be damaging non-synonymous DNV was identified. Some cases (e.g., B06, B08, and B13) were found to carry more than one damaging DNVs. Clinical phenotypes of the NTDs in the 13 trios are shown in Supplementary Table S2.

**TABLE 1:** *De novo* variants identified in the 13 studied anencephaly trios.

**Sample**	**Gene**	**pLI**	**ExonicFunc**	**DNA&AA change**	**SIFT^∗^**	**PolyPhen2^∗^**
B03	*SATB2*	1	Non-synonymous SNV	NM_001172509:c.C1594T:p.R532C	D	D
B03	*ACRBP*	0	Synonymous SNV	NM_032489:c.C231T:p.D77D	NA	NA
B05	*ORC3*	0	Non-synonymous SNV	NM_001197259:c.C868G:p.R290G	D	D
B06	*SPHKAP*	0.94	Frameshift deletion	NM_001142644:c.2629_2633del:p.877_878del	NA	NA
B06	*FBXO8*	0.49	Synonymous SNV	NM_012180:c.A177G:p.A59A	NA	NA
B06	*PPARGC1B*	0.5	Non-synonymous SNV	NM_001172698:c.G2281A:p.G761S	D	B
B07	*WIPI1*	0	Non-synonymous SNV	NM_017983:c.G983A:p.R328Q	D	D
B08	*DMXL2*	1	Non-synonymous SNV	NM_001174117:c.C6470T:p.T2157M	D	B
B08	*NCOR1*	1	Stopgain SNV	NM_001190440:c.T5721A:p.Y1907X	NA	NA
B11	*BRF1*	0	Non-synonymous SNV	NM_001242789:c.G1001A:p.R334Q	T	D
B13	*FOXE3*	0.18	Non-frameshift deletion	NM_012186:c.179_181del:p.60_61del	NA	NA
B13	*SHPRH*	0	Non-synonymous SNV	NM_001042683:c.A515G:p.D172G	D	B
B12	*CNOT3*	1	Non-synonymous SNV	NM_014516:c.AC626-6277TA:p.Y209F	T	D

*^∗^SIFT prediction (cutoff 0.05), PolyPhen prediction (cutoff 0.05); B: Benign; D: damaging; T: tolerant; NA: not available.*

### Variants of *WIPI1* Gene in a NTD Cohort

As we previously found that WIPI1 knockdown in zebrafish causes convergent extension defect, and WD repeat protein (encoded by *WIPI1* gene) plays an important role in the autophagy pathway which is considered to be essential for neural tube closure (Fimia et al., 2007; Xu et al., 2013), we further determined whether variants of *WIPI1* gene increase NTD risk in a large cohort. Clinical phenotype of NTDs in the cohort of 502 NTD cases was shown in Supplementary Table S3. We used next-generation sequencing to detect variants in the *WIPI1* gene in all NTD cases. We found another three variants in the *WIPI1* gene in four cases in the NTD cohort. We summarized the variants within *WIPI1* coding sequence identified in NTD-affected trios and in NTD cohort in Table 2. T418M variant was identified in two cases, one with both anencephaly and spina bifida, and one with only spina bifida. An additional two cases with anencephaly carried G313R and L406P variants, respectively. Amino acid conservation analysis showed all the variants located at highly conserved nucleotides (Supplementary Figure S2). Compared with East Asia WIPI1 variants in gnomAD database, there is significant (OR: 3.07, 95% CI: 1.07–8.84, Fisher’s exact test *P*-value: 0.053) enrichment of WIPI1 rare damaging missense variants in anencephaly. In addition, the DNV has been reported in the Clinvar^6^.

**TABLE 2:** Variants within WIPI1 coding sequence identified in the NTD cases.

**Amino acid position^a^**	**Exon**	**Chr**	**Position**	**Nucleotide change^b^**	**dbSNP reference number**	**ExAC**	**ExAC East Asian**	**Alt allele frequency**	**SIFT prediction^c^**	**PolyPhen prediction^c^**	**PROVEAN prediction^c^**
p.R328Q	exon10	chr17	66425060	c.983G>A	rs146357218	0.0000252	0.000234	0.00190114	Damaging	Damaging	Deleterious
p.G313R	exon9	chr17	66426165	c.937G>A	rs771913875	0.0000494	0	0.00325380	Damaging	Damaging	Deleterious
p.T418M	exon12	chr17	66422256	c.1253C>T	rs564818335	0.0000412	0	0.00106157	Tolerated	Probably damaging	Neutral
p.L406P	exon12	chr17	66422292	c.1217T>C	rs77156594	0.001164	0.001735	0.00106610	Tolerated	Benign	Neutral

*^*a*^Reference protein sequence NP_060453.3. ^*b*^Nucleotide numbering reflects + 1 corresponding to the A of ATG, mRNA sequence NM_017983. ^*c*^SIFT prediction (cutoff 0.05), PolyPhen prediction (cutoff 0.85), and PROVEAN prediction (cutoff −2.5).*

### *wipi1* Variants Affect Convergent Extension in Zebrafish

**FIGURE 2:**
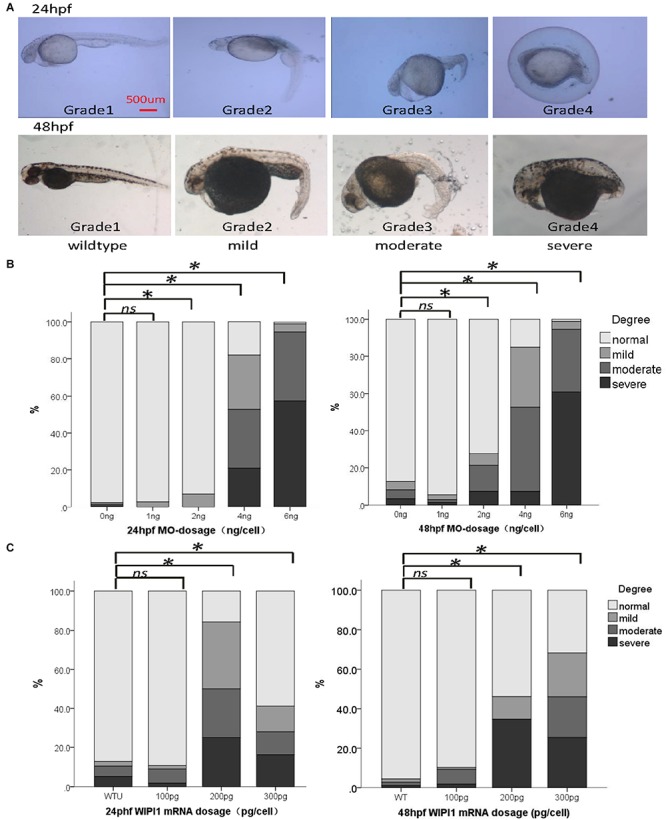
Abnormal expression of *wipi1* affected zebrafish embryo convergent extension. **(A)** Zebrafish embryos injected with *wipi1-*MO (antisense morpholino) show defects of convergent extension, including shortening of the trunk, and crooked or bent tails. To assess the extension defects quantitatively, the phenotypic classes were scored as grade 1 (normal), grade 2 (mild), grade 3 (moderate), and grade 4 (severe) in zebrafish embryos at 24 and 48 h post-fertilization (hpf) (scale bar = 500 μm). **(B)** Zebrafish embryos injected with *wipi1*-MO exhibit dosage-dependent convergent extension deficits at 24 and 48 hpf. Injection with *wipi1*-MO at dose of 2–6 ng showed a significant difference compared with control injection. **(C)** Overexpression of Wipi1 in zebrafish embryos at 24 and 48 hpf. Injection 200 pg mRNA and 300 pg mRNA resulted in a significant increase of malformed zebrafish. There was no correlation between mRNA dosage and severity of malformation (^∗^*P* < 0.05).

To examine the potential pathogenic effect of these *wipi1* variants *in vivo*, we investigated their functions on convergent extension in zebrafish model which represents a well-established model for genetic variation studies on NTDs (Roszko et al., 2009; Reynolds et al., 2010). Knockdown or overexpression of NTD-related genes in zebrafish leads to a defective convergent extension manifested mainly by shortened body axis (Roszko et al., 2009; Reynolds et al., 2010). Embryos with convergent extension defects had shorter anterior/posterior axes, as well as crooked or bent tails which are commonly considered to be indications of an NTD (Figure 2A). We clustered these injected embryos into four categories according to the severity of their morphology: grade 1, WT like; grade 2, mild defect, up to one-forth shortened axis compared with WT embryos; grade 3, moderate, up to 1/2 shortened axis; grade 4, severe, the body axis does not extend out of the range of the yolk ball (Figure 2A). We examined the distributions of these four categories among different treatment groups.

Either the knockdown or overexpression of WT *wipi1* caused convergent extension defects with a marked shortening of the anterior–posterior axis (Figures 2B,C). As shown in Figure 2B, the distribution of the phenotype was significantly different in embryos injected with different MO doses (1, 2, 4, and 6 ng) of *wipi1* antisense MO at 24 and 48 hpf (24 h: χ^2^ = 932.8, *P* < 0.001; 48 h: χ^2^ = 606.7, *P* < 0.001). The dose–response relationship was observed (*P* for trend < 0.05) and the rate of abnormal morphology increased dramatically in embryos injected with 4 ng of *wipi1-*MO. We chose 4 ng as the optimal injection dose for all subsequent tests.

Embryos injected with different concentrations of wild-type mRNA also presented with mild to severe malformations at 24 and 48 hpf (Figure 2C). Compared with the control group, the rate of abnormal morphology increased in embryos injected with 200–300 pg mRNA (*P* < 0.05), but not in embryos injected with 100 pg mRNA at 24 and 48 hpf. The dose–response relationship was observed in the embryos at 48 hpf (*P* for trend < 0.05).

**FIGURE 3:**
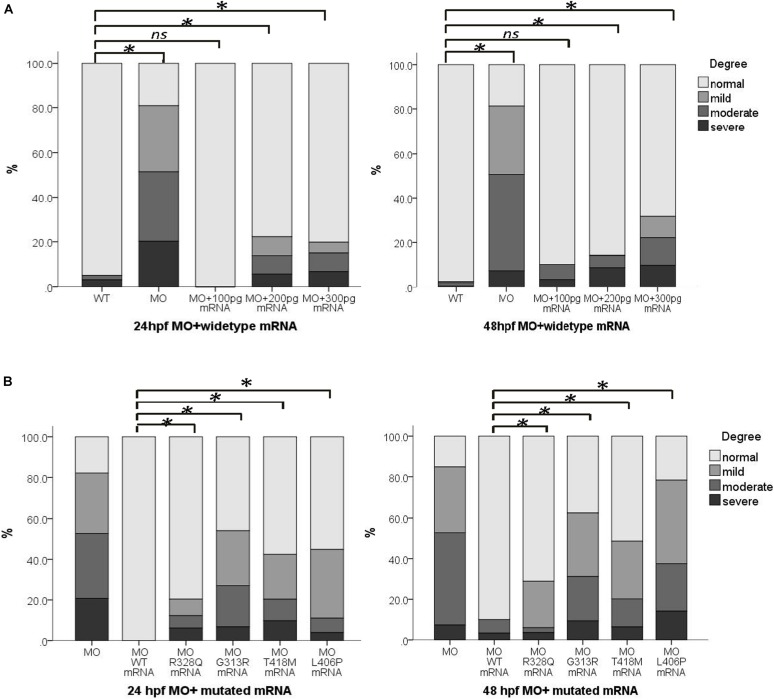
*wipi1* mRNA variants affected the convergent extension in zebrafish embryos treated with *wipi1*-MO. **(A)** *WIPI1* WT mRNA of 100–300 pg rescued the convergent extension defects induced by co-injection of *wipi1*-MO in zebrafish embryos at 24 and 48 hpf. There was no difference in malformation between embryos co-treated with 100 pg mRNA and *wipi1*-MO dosage and control embryos untreated with MO or mRNA. **(B)** *WIPI1* mRNA co-injected with *wipi1*-MO rescued the convergent extension. However, *WIPI1* R328Q, G313R, T418M, or L406P mRNA injection failed to rescue the phenotype, while *WIPI1* WT mRNA injection compensated for the phenotype (^∗^*P* < 0.05).

To further clarify the function of *wipi1*, we carried out rescue experiment. We first tested different concentrations (100–300 pg) of wild-type *WIPI1* mRNA co-injected with 4 ng *wipi1*-MO (Figure 3A). Compared with the MO group, the rate of abnormal morphology decreased in embryos injected with 100–300 pg oF *WIPI1* wild-type mRNA (*P* < 0.05). Compared with control group, embryos injected with 100 pg of *WIPI1* wild-type mRNA had a similar response frequency of abnormal embryos (*P* > 0.05), suggesting a good rescue effect.

We co-injected 4 ng *wipi1*-MO with each *wipi1* variant mRNA to clarify the effect of these *wipi1* variants (Figure 3B). All the *wipi1* mutants could not reverse the phenotype of the *wipi1* knock-down and only manifested mild effects, whereas WT-MO dramatically ameliorated the phenotype at 24 and 48 hpf (*P* < 0.05). The malformation response frequency of the Wipi1 L406P variant mRNA-MO co-injected group was significantly higher than other co-injected groups (*P* < 0.05), and showed no difference from the MO group (*P* = 0.130), indicating that this treatment was the least effective in rescuing the normal phenotype among all the mutants at 48 hpf (*P* < 0.05).

### *WIPI1* Variants Affect Expression

**FIGURE 4:**
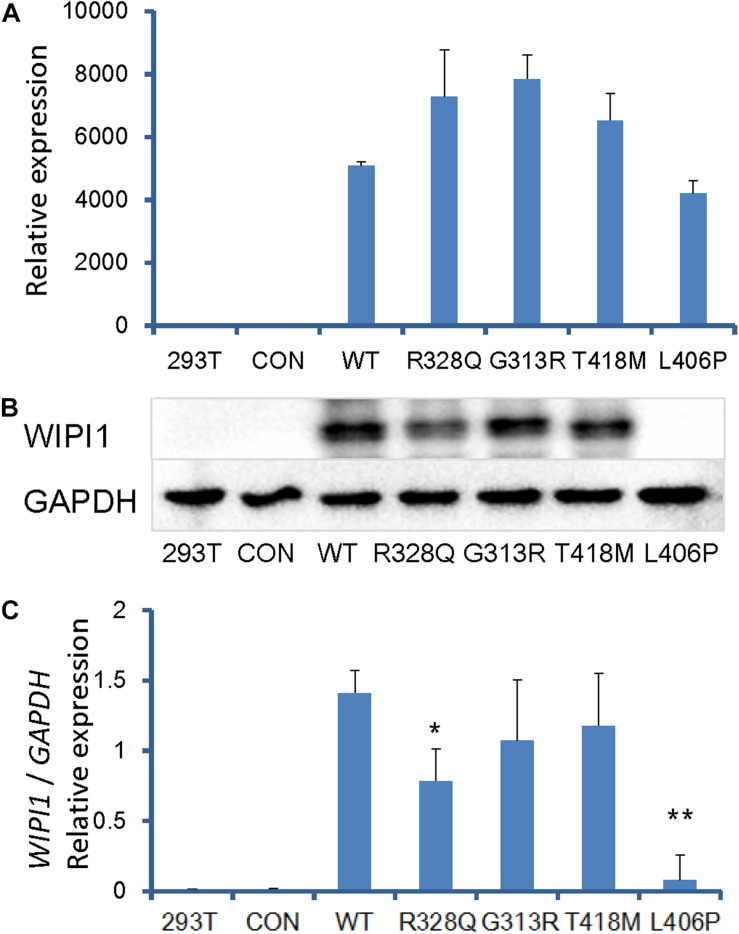
*WIPI1* variants and their expression of mRNA and protein. Human HEK 293 cells were cotransfected with pIRES2-dsRed and *WIPI1* variants. At 48 h post-transfection, the cells were harvested for real-time PCR and Western blotting. **(A)** *WIPI1* mRNA expression level was not influenced by WIPI1 R328Q, G313R, T418M, or L406P mutations. **(B)** *WIPI1* L406P mutation affected WIPI1 protein expression, while *WIPI1* R328Q, G313R, or T418M had no adverse effects on WIPI1 protein expression. **(C)** Relative WIPI1 mutant protein expression analysis. The data were derived from three independent experiments and expressed as mean ± SD. Data for *WIPI1* mRNA and expression were normalized by *GAPDH* for each sample. Error bars represent standard error/deviation of the mean. The asterisk indicates a statistically significant difference between wild-type and L406P mutation (^∗∗^*P* < 0.01; ^∗^*P* < 0.05).

To investigate the effect of human *WIPI1* mutants on the expression of mRNA and protein, we transfected with dsRed-tagged *WIPI1* wild-type, R328Q, G313R, T418M, or L406P variant into HEK293T cells. No significant difference in transfection efficiency was detected between wild-type and the mutant constructs (Supplementary Figure S3). *WIPI1* mRNA expression level was not influenced by WIPI1 R328Q, G313R, T418M, or L406P variant (Figure 4A). However, *WIPI1* L406P variant completely affected while the *WIPI1* R328Q variant partially affected WIPI1 protein expression, and the *WIPI1* G313R or T418M variants had no effects on WIPI1 protein expression (Figures 4B,C).

### Subcellular Localization

We examined the WIPI1 subcellular localization of the four NTD case-specific missense variants with the wild-type localization serving as a positive control. All of the four variants localized to the cell’s cytoplasm and maintained the same subcellular localization pattern as did the wild-type (Supplementary Figure S4).

## Discussion

In this study, we performed WES on 13 parent–offspring trios where the child was diagnosed with anencephaly, resulting in the identification of a total of 13 coding DNVs in 8 cases. The identified non-synonymous SNVs were all predicted to be damaging DNVs by either SIFT or PolyPhen2. This indicates that WES on trios is a robust strategy to identify potential causative variants for anencephaly. Two LoF DNVs were identified in pLI > 0.9 genes, *SPHKAP* and *NCOR1*. SPHKAP plays role in cAMP signal pathway which may interact with NTD-related G-protein-coupled receptors such as GPR161 (Matteson et al., 2008; Mukhopadhyay et al., 2013; Kim et al., 2019) and CELSR1 (Robinson et al., 2012; Lei et al., 2014). NCOR1 is a nuclear receptor corepressor which was previously associated with Rett Syndrome (Lyst et al., 2013) and Endometrial Hyperplasia (Kashima et al., 2009). NCOR1 plays role in a large corepressor complex which is associated with the retinoid acid receptor (RAR) in the absence of ligand. It is well known that overdose retinoid acid can cause NTDs (Liu et al., 2018). Therefore, *NCOR1* variants could increase NTD risk through RAR pathway.

We subsequently identified three variants in the *WIPI1* gene using exome sequencing strategies in an independent population consisting of 502 NTD cases. We performed functional studies by combining the evidence obtained using knockdown/rescue assays in a zebrafish model and transfection expression studies in HEK293T cells. Our findings suggest that functional variants in *WIPI1* gene might contribute to the etiology of human anencephaly.

Although DNVs have been shown to contribute to a range of rare and common birth defect phenotypes, including congenital heart defects and autism (Sanders et al., 2012; Veltman and Brunner, 2012; Zaidi et al., 2013), few studies have been published utilizing either WES or WGS in patients with spina bifida or anencephaly to identify the recurrent DNVs that contribute to their etiology. The only WES study involving NTD cases was conducted by Lemay et al. (2015, 2019) in Canada. As for the anencephaly subtype, only eight trios were recruited. This study identified one LoF DNV in *SHROOM3* gene, which was assumed to be associated with the development of anencephaly (Lemay et al., 2015, 2019). In the present study, we conducted a WES analysis in 13 parent–offspring trios involving a child with anencephaly. In one anencephaly case without visible cardiac defects, two nucleotide variants led to a single amino acid change were found in CNOT3, which was previously associated with cardiac defects in fly or mouse models, and with embryonical lethality in Cnot3-null mice (Neely et al., 2010). This is the first study reporting WES for NTDs in Chinese Han populations, which provides an unbiased description of the genetic variation allowing us to explore for the first time the full allele frequency spectrum of anencephaly cases.

We further examined variants of the *WIPI1* gene in a large NTD cohort, considering that WD repeat protein (encoded by *WIPI1* gene) has been previously linked to the autophagy pathway, which has a protective function against the onset of neurodegeneration and may well be essential for normal neural tube closure (Fimia et al., 2007; Xu et al., 2013). Resequencing 502 Chinese NTDs cases, we identified three candidate disease-related *WIPI1* variants (p.G313R, p.T418M, and p.L406P). All of the variants are heterozygous and two were private. No any additional known variants in PCP genes related to NTDs were found in cases carried these three WIPI1 variants (Wang et al., 2018). Unfortunately, due to the absence of corresponding parental samples, we could not confirm their inheritance pattern. It is interesting to note that all the three variants were from anencephaly cases, except p.T418M which was identified in a spina bifida case, although the number of anencephalic fetuses we examined was significantly less than that of spina bifida cases. On the other hand, because p.T418M was not shown to be a functional variant, this variant may not be causal for spina bifida or encephalocele. This suggests that cases with anencephaly may be more likely to carry *WIPI1* variants than cases with either spina bifida or encephalocele. Future studies of gene knock-out animal models should help to determine whether the *WIPI1* variants are specific to cranial NTDs, which could have important implications for the etiology of NTDs.

Functional validation of these mutants represents an essential step toward dissecting the etiological complexity of the human anencephaly phenotype, and investigating the underlying its pathogenic mechanisms. According to the alignment in species and amino acid conservation analysis, the four variants observed in our study were highly conserved residues. Subcellular localization showed that the four variants didn’t affect the subcellular distribution of the WIPI1 protein. To investigate the effect of these variants on gene expression, we transfected with dsRed-tagged *WIPI1* wild-type and variants into HEK293T cells. Western blotting revealed that the p.L406P variant was harmful to the stability of the *WIPI1* protein, while p.R328Q was only moderately harmful.

We further investigated potential functional properties of the *WIPI1* variants in zebrafish model which is a well-established model for genetic variation studies on NTDs (Roszko et al., 2009; Reynolds et al., 2010). Convergent extension in zebrafish is a polarized cellular rearrangement that leads to the narrowing of the mediolateral axis and lengthening of the anteroposterior axis for gastrulation and neural tube formation. Our observations of convergent extension in zebrafish rescue assays showed a failure to rescue with the transcripts of human *WIPI1* p.L406P and p.R328Q variants. These data indicate that the human RNA containing the p.L406P and p.R328Q variants fails to function as well as wild-type RNA, suggesting that the two variants impair the protein function during embryogenesis and might be hypomorphs. A raised concern is that the p.L406P and p.R328Q variants were rarely observed in the Exome Aggregation Consortium (ExAC) database. We previously found that genetic variants might interact in a digenic fashion to generate the visible NTD phenotypes (Wang et al., 2018). There might be other potential variations in the *WIPI1* gene or other genes that contribute to compound heterozygous genotypes that lead to NTD phenotypes. To further dissect the roles of these variations and the *WIPI1* gene during embryogenic development, additional functional studies using mouse conditional knockouts are needed.

## Conclusion

In conclusion, *de novo* damaging variants are the main culprit for majority of anencephalic cases. Missense variants in *WIPI1* may play a role in the genetic etiology of anencephaly, and LoF variants in *SPHKAP* and *NCOR1* may also contribute to anencephaly. These findings expand our collective knowledge of the genetic mechanisms contributing to NTDs, and strongly implicate *de novo* damaging variants in the etiology of anencephaly. Our study also highlights the importance of WES or further whole-genome scale variant screens in a large number of patients to discover new genes and new variants that cause human congenital defects.

## Data Availability Statement

The datasets for this manuscript are not publicly available because of ethical and legal reasons. Requests to access the datasets should be directed to AR at renag@bjmu.edu.cn.

## Ethics Statement

The studies involving human participants were reviewed and approved by the Institutional Review Board of Peking University. The patients/participants provided their written informed consent to participate in this study.

## Author Contributions

AR, RF, and YL conceptualized the study. LW supervised the implementation, helped data analysis, and drafted the manuscript. TT, YS, and BZ conducted the zebrafish study. NL performed the bioinformatic data analysis. YL helped the study design and data interpretation. PZ performed the pipeline sensitivity and specificity test. XC provided critical comments on the earlier version of the manuscript. TT participated in the functional study. LW, AR, LJ, and ZL participated in subject enrollment. AR and RF critically revised the manuscript.

## Conflict of Interest

The authors declare that the research was conducted in the absence of any commercial or financial relationships that could be construed as a potential conflict of interest.

## Footnotes

1 https://github.com/genome-in-a-bottle

2 http://genetics.bwh.harvard.edu/pph2/

3 http://sift.bii.a-star.edu.sg/

4 http://provean.jcvi.org/index.php

5 https://gnomad.broadinstitute.org/

6 https://www.ncbi.nlm.nih.gov/clinvar/

## References

[B1] Allache R. De Marco P. Merello E. Capra V. Kibar Z. (2012). Role of the planar cell polarity gene CELSR1 in neural tube defects and caudal agenesis. *Birth Defects Res. A Clin. Mol. Teratol.* 94 176–181. 10.1002/bdra.23002 22371354

[B2] Bassuk A. G. Kibar Z. (2009). Genetic basis of neural tube defects. *Semin. Pediatr. Neurol.* 16 101–110. 10.1016/j.spen.2009.06.001 19778707

[B3] Blom H. J. Shaw G. M. den Heijer M. Finnell R. H. (2006). Neural tube defects and folate: case far from closed. *Nat. Rev. Neurosci.* 7 724–731. 10.1038/nrn1986 16924261 PMC2970514

[B4] Bosoi C. M. Capra V. Allache R. Trinh V. Q. De Marco P. Merello E. (2011). Identification and characterization of novel rare mutations in the planar cell polarity gene PRICKLE1 in human neural tube defects. *Hum. Mutat.* 32 1371–1375. 10.1002/humu.21589 21901791 PMC3217084

[B5] Chen Z. Lei Y. Zheng Y. Aguiar-Pulido V. Ross M. E. Peng R. (2018). Threshold for neural tube defect risk by accumulated singleton loss-of-function variants. *Cell Res.* 28 1039–1041. 10.1038/s41422-018-0061-63 29976953 PMC6170406

[B6] De Marco P. Merello E. Consales A. Piatelli G. Cama A. Kibar Z. (2013). Genetic analysis of disheveled 2 and disheveled 3 in human neural tube defects. *J. Mol. Neurosci.* 49 582–588. 10.1007/s12031-012-9871-9879 22892949 PMC3566388

[B7] De Marco P. Merello E. Rossi A. Piatelli G. Cama A. Kibar Z. (2012). FZD6 is a novel gene for human neural tube defects. *Hum. Mutat.* 33 384–390. 10.1002/humu.21643 22045688 PMC3482927

[B8] Fimia G. M. Stoykova A. Romagnoli A. Giunta L. Di Bartolomeo S. Nardacci R. (2007). Ambra1 regulates autophagy and development of the nervous system. *Nature* 447 1121–1125. 10.1038/nature05925 17589504

[B9] Girard S. L. Gauthier J. Noreau A. Xiong L. Zhou S. Jouan L. (2011). Increased exonic de novo mutation rate in individuals with schizophrenia. *Nat. Genet.* 43 860–863. 10.1038/ng.886 21743468

[B10] Gray R. S. Roszko I. Solnica-Krezel L. (2011). Planar cell polarity: coordinating morphogenetic cell behaviors with embryonic polarity. *Dev. Cell* 21 120–133. 10.1016/j.devcel.2011.06.011 21763613 PMC3166557

[B11] Haddow J. E. (2011). Folic acid and neural tube defects. *Genet. Med.* 13:849. 10.1097/GIM.0b013e3182326e9d 21885925

[B12] Harris M. J. Juriloff D. M. (2007). Mouse mutants with neural tube closure defects and their role in understanding human neural tube defects. *Birth Defects Res. A Clin. Mol. Teratol.* 79 187–210. 10.1002/bdra.20333 17177317

[B13] Harris M. J. Juriloff D. M. (2010). An update to the list of mouse mutants with neural tube closure defects and advances toward a complete genetic perspective of neural tube closure. *Birth Defects Res. A Clin. Mol. Teratol.* 88 653–669. 10.1002/bdra.20676 20740593

[B14] Juriloff D. M. Harris M. J. (2012). A consideration of the evidence that genetic defects in planar cell polarity contribute to the etiology of human neural tube defects. *Birth Defects Res. A Clin. Mol. Teratol.* 94 824–840. 10.1002/bdra.23079 23024041

[B15] Kang Y. Ding H. Zhou H. X. Wei Z. J. Liu L. Pan D. Y. (2018). Epidemiology of worldwide spinal cord injury: a literature review. *J. Neurorestoratol.* 6 1–9. 10.2147/jn.S143236

[B16] Kashima H. Horiuchi A. Uchikawa J. Miyamoto T. Suzuki A. Ashida T. (2009). Up-regulation of nuclear receptor corepressor (NCoR) in progestin-induced growth suppression of endometrial hyperplasia and carcinoma. *Anticancer. Res.* 29 1023–1029. 19414341

[B17] Kibar Z. Capra V. Gros P. (2007a). Toward understanding the genetic basis of neural tube defects. *Clin. Genet.* 71 295–310. 10.1111/j.1399-0004.2007.00793.x 17470131

[B18] Kibar Z. Torban E. McDearmid J. R. Reynolds A. Berghout J. Mathieu M. (2007b). Mutations in VANGL1 associated with neural-tube defects. *N. Engl. J. Med.* 356 1432–1437. 10.1056/NEJMoa060651 17409324

[B19] Kim S. E. Lei Y. Hwang S. H. Wlodarczyk B. J. Mukhopadhyay S. Shaw G. M. (2019). Dominant negative GPR161 rare variants are risk factors of human spina bifida. *Hum. Mol. Genet.* 28 200–208. 10.1093/hmg/ddy339 30256984 PMC6321953

[B20] Lei Y. Zhu H. Yang W. Ross M. E. Shaw G. M. Finnell R. H. (2014). Identification of novel CELSR1 mutations in spina bifida. *PLoS One* 9:e92207. 10.1371/journal.pone.0092207 24632739 PMC3954890

[B21] Lei Y. P. Zhang T. Li H. Wu B. L. Jin L. Wang H. Y. (2010). VANGL2 mutations in human cranial neural-tube defects. *N. Engl. J. Med.* 362 2232–2235. 10.1056/NEJMc0910820 20558380

[B22] Lemay P. De Marco P. Traverso M. Merello E. Dionne-Laporte A. Spiegelman D. (2019). Whole exome sequencing identifies novel predisposing genes in neural tube defects. *Mol. Genet. Genomic Med.* 7:e00467. 10.1002/mgg3.467 30415495 PMC6382446

[B23] Lemay P. Guyot M. C. Tremblay E. Dionne-Laporte A. Spiegelman D. Henrion E. (2015). Loss-of-function de novo mutations play an important role in severe human neural tube defects. *J. Med. Genet.* 52 493–497. 10.1136/jmedgenet-2015-103027 25805808

[B24] Liu D. Xue J. Liu Y. Gu H. Wei X. Ma W. (2018). Inhibition of NRF2 signaling and increased reactive oxygen species during embryogenesis in a rat model of retinoic acid-induced neural tube defects. *Neurotoxicology* 69 84–92. 10.1016/j.neuro.2018.09.005 30267739

[B25] Liu J. Zhang L. Li Z. Jin L. Zhang Y. Ye R. (2016). Prevalence and trend of neural tube defects in five counties in Shanxi province of Northern China, 2000 to 2014. *Birth Defects Res. A Clin. Mol. Teratol.* 106 267–274. 10.1002/bdra.23486 26879384

[B26] Lyst M. J. Ekiert R. Ebert D. H. Merusi C. Nowak J. Selfridge J. (2013). Rett syndrome mutations abolish the interaction of MeCP2 with the NCoR/SMRT co-repressor. *Nat. Neurosci.* 16 898–902. 10.1038/nn.3434 23770565 PMC3786392

[B27] Manolio T. A. Collins F. S. Cox N. J. Goldstein D. B. Hindorff L. A. Hunter D. J. (2009). Finding the missing heritability of complex diseases. *Nature* 461 747–753. 10.1038/nature08494 19812666 PMC2831613

[B28] Matteson P. G. Desai J. Korstanje R. Lazar G. Borsuk T. E. Rollins J. (2008). The orphan G protein-coupled receptor, Gpr161, encodes the vacuolated lens locus and controls neurulation and lens development. *Proc. Natl. Acad. Sci. U.S.A.* 105 2088–2093. 10.1073/pnas.0705657105 18250320 PMC2538885

[B29] Mukhopadhyay S. Wen X. Ratti N. Loktev A. Rangell L. Scales S. J. (2013). The ciliary G-protein-coupled receptor Gpr161 negatively regulates the Sonic hedgehog pathway via cAMP signaling. *Cell* 152 210–223. 10.1016/j.cell.2012.12.026 23332756

[B30] Murdoch J. N. Damrau C. Paudyal A. Bogani D. Wells S. Greene N. D. (2014). Genetic interactions between planar cell polarity genes cause diverse neural tube defects in mice. *Dis. Model. Mech.* 7 1153–1163. 10.1242/dmm.016758 25128525 PMC4174526

[B31] Nava C. Dalle C. Rastetter A. Striano P. de Kovel C. G. Nabbout R. (2014). De novo mutations in HCN1 cause early infantile epileptic encephalopathy. *Nat. Genet.* 46 640–645. 10.1038/ng.2952 24747641

[B32] Neale B. M. Kou Y. Liu L. Ma’ayan A. Samocha K. E. Sabo A. (2012). Patterns and rates of exonic de novo mutations in autism spectrum disorders. *Nature* 485 242–245. 10.1038/nature11011 22495311 PMC3613847

[B33] Neely G. G. Kuba K. Cammarato A. Isobe K. Amann S. Zhang L. (2010). A global in vivo Drosophila RNAi screen identifies NOT3 as a conserved regulator of heart function. *Cell* 141 142–153. 10.1016/j.cell.2010.02.023 20371351 PMC2855221

[B34] Rauch A. Wieczorek D. Graf E. Wieland T. Endele S. Schwarzmayr T. (2012). Range of genetic mutations associated with severe non-syndromic sporadic intellectual disability: an exome sequencing study. *Lancet* 380 1674–1682. 10.1016/S0140-6736(12)61480-61489 23020937

[B35] Reynolds A. McDearmid J. R. Lachance S. De Marco P. Merello E. Capra V. (2010). VANGL1 rare variants associated with neural tube defects affect convergent extension in zebrafish. *Mech. Dev.* 127 385–392. 10.1016/j.mod.2009.12.002 20043994 PMC2965831

[B36] Riviere J. B. van Bon B. W. Hoischen A. Kholmanskikh S. S. O’Roak B. J. Gilissen C. (2012). De novo mutations in the actin genes ACTB and ACTG1 cause Baraitser-Winter syndrome. *Nat. Genet.* 44 440–444. 10.1038/ng.1091 22366783 PMC3677859

[B37] Robinson A. Escuin S. Doudney K. Vekemans M. Stevenson R. E. Greene N. D. (2012). Mutations in the planar cell polarity genes CELSR1 and SCRIB are associated with the severe neural tube defect craniorachischisis. *Hum. Mutat.* 33 440–447. 10.1002/humu.21662 22095531 PMC4772123

[B38] Roszko I. Sawada A. Solnica-Krezel L. (2009). Regulation of convergence and extension movements during vertebrate gastrulation by the Wnt/PCP pathway. *Semin. Cell Dev. Biol.* 20 986–997. 10.1016/j.semcdb.2009.09.004 19761865 PMC2796982

[B39] Sanders S. J. Murtha M. T. Gupta A. R. Murdoch J. D. Raubeson M. J. Willsey A. J. (2012). De novo mutations revealed by whole-exome sequencing are strongly associated with autism. *Nature* 485 237–241. 10.1038/nature10945 22495306 PMC3667984

[B40] Schwarze K. Buchanan J. Taylor J. C. Wordsworth S. (2018). Are whole-exome and whole-genome sequencing approaches cost-effective? A systematic review of the literature. *Genet. Med.* 20 1122–1130. 10.1038/gim.2017.247 29446766

[B41] Seo J. H. Zilber Y. Babayeva S. Liu J. Kyriakopoulos P. De Marco P. (2011). Mutations in the planar cell polarity gene, Fuzzy, are associated with neural tube defects in humans. *Hum. Mol. Genet.* 20 4324–4333. 10.1093/hmg/ddr359 21840926

[B42] Simons M. Mlodzik M. (2008). Planar cell polarity signaling: from fly development to human disease. *Annu. Rev. Genet.* 42 517–540. 10.1146/annurev.genet.42.110807.091432 18710302 PMC2814158

[B43] Thorvaldsdottir H. Robinson J. T. Mesirov J. P. (2013). Integrative Genomics Viewer (IGV): high-performance genomics data visualization and exploration. *Brief Bioinform.* 14 178–192. 10.1093/bib/bbs017 22517427 PMC3603213

[B44] Veltman J. A. Brunner H. G. (2012). De novo mutations in human genetic disease. *Nat. Rev. Genet.* 13 565–575. 10.1038/nrg3241 22805709

[B45] Wallingford J. B. Niswander L. A. Shaw G. M. Finnell R. H. (2013). The continuing challenge of understanding, preventing, and treating neural tube defects. *Science* 339:1222002. 10.1126/science.1222002 23449594 PMC3677196

[B46] Wang L. Xiao Y. Tian T. Jin L. Lei Y. Finnell R. H. (2018). Digenic variants of planar cell polarity genes in human neural tube defect patients. *Mol. Genet. Metab.* 124 94–100. 10.1016/j.ymgme.2018.03.005 29573971 PMC5966321

[B47] Wilde J. J. Petersen J. R. Niswander L. (2014). Genetic, epigenetic, and environmental contributions to neural tube closure. *Annu. Rev. Genet.* 48 583–611. 10.1146/annurev-genet-120213-192208 25292356 PMC4649936

[B48] Xu C. Li X. Wang F. Weng H. Yang P. (2013). Trehalose prevents neural tube defects by correcting maternal diabetes-suppressed autophagy and neurogenesis. *Am. J. Physiol. Endocrinol. Metab.* 305 E667–E678. 10.1152/ajpendo.00185.2013 23880312 PMC3761168

[B49] Yates C. L. Monaghan K. G. Copenheaver D. Retterer K. Scuffins J. Kucera C. R. (2017). Whole-exome sequencing on deceased fetuses with ultrasound anomalies: expanding our knowledge of genetic disease during fetal development. *Genet. Med.* 19 1171–1178. 10.1038/gim.2017.31 28425981

[B50] Zaidi S. Choi M. Wakimoto H. Ma L. Jiang J. Overton J. D. (2013). De novo mutations in histone-modifying genes in congenital heart disease. *Nature* 498 220–223. 10.1038/nature12141 23665959 PMC3706629

[B51] Zook J. M. Catoe D. McDaniel J. Vang L. Spies N. Sidow A. (2016). Extensive sequencing of seven human genomes to characterize benchmark reference materials. *Sci. Data* 3:160025. 10.1038/sdata.2016.25 27271295 PMC4896128

