# Supplementary material for: Whole-Exome Sequencing Identifies Damaging de novo Variants in Anencephalic Cases
Source: Front Neurosci. 2019 Nov 29;13:1285. doi: 10.3389/fnins.2019.01285 (PMC6896715; doi:10.3389/fnins.2019.01285)
Supplement: Supplementary file 1 [file Table_1.docx]

## SUPPLEMENTARY MATERIALS

### METHODS

**Whole-exome data analysis**

We used the Burrows-Wheeler Aligner (BWA) to align sequence reads to the human reference genome (hg19) reference genome and removed potential duplicate paired-end reads. SAM formatted file was converted into BAM file with samtools. We used picard to sort BAM, mark duplicates, add headers and reorder the BAM. The Genome Analysis Toolkit (GATK) was used to recalibrate base quality scores, realign around indels and mark duplicate reads. We called variants (both snp and indel) using modules haplotype caller (HC) and made joint genotyping with genotypeGVCFs module. The sequencing analysis and variant calling were according to the literature (PMIDs: 22366783, 23665959, 27479907). Variants with quality scores < 30, allele balance > 0.75, sequencing depth < 4, quality/depth ratio < 5.0, length of homopolymer run > 5.0, and strand bias > −0.10 were flagged and excluded from subsequent analyses. Coverage was assessed with the GATK Depth of Coverage tool by ignoring reads with mapping quality < 20 and bases with base quality < 30. On average our samples had a median coverage of 80X (SD =18X) across targeted regions. 88% of targeted bases read had at least 30X coverage. The summary of sequencing QA/QC is listed in Table S1.

We prioritized rare functional variants (silent, splice site, missense, frameshift, or stop gain and stop lost) that were heterozygous *de novo* variants, homozygous, or compound heterozygous in the child and excluded variants with a Minor Allele Frequency (MAF) >0.01 in dbSNP132, in the Exome Variant Server (http://evs.gs.washington.edu/EVS/), 1000 Genomes Project (http://www.1000genomes.org/), or Exome Aggregation Consortium database (ExAC), Cambridge, MA(<http://exac.broadinstitute>. org).

### Multiplex PCR amplification and next-generation sequencing

Multiplex PCR amplification and next-generation sequencing was performed to screen for DNA variants along the entire coding regions and intron-exon boundaries of the targeted *WIPI1* gene. Primers were designed using primer5. A total of 14 oligonucleotide pairs were constructed to cover all of the coding sequences and intron-exon boundaries of the *WIPI1* gene. After the first round of primer design, under the most stringent conditions (no SNPs in primer annealing region, amplicon length between 200-270bp, GC content between 30 and 80%), the 14 oligonucleotide pairs were put into one multiplex PCR panels that amplified all of the target regions. The amplification reactions were carried out on an AB 2720 Thermal Cycler (Life Technologies Corporation, USA). The PCR product of each sample was labeled with 8 bp barcode; all the libraries of each sample were pooled. After cluster generation and hybridization of sequencing primer, base incorporation was carried out on a MiSeq Benchtop Sequencer (Illumina, Inc, San Diego, CA) in one single lane following the manufacturer's standard cluster generation and sequencing protocols. The sequencing reactions ran for 300 cycles per read to generate paired-end reads including 300 bp at each end and 8 bp of the index tag.

### Supplementary Table S1 Sequencing QA/QC for samples

| Category | Samples |
| --- | --- |
| # of reads per sample (M) | 93.6 ± 24.2 |
| Median coverage at each targeted base (X) | 80.2 ± 18.2 |
| Mean coverage at each targeted base (X) | 97 ± 25.1 |
| % of all bases that map to human genome | 90.3% ± 0.8 |
| % of all bases that map to target | 68.1% ± 4.5 |
| % of targeted bases read at least 8x | 96.0% ± 1 |
| % of targeted bases read at least 20x | 91.4% ± 3.4 |
| % of targeted bases read at least 30x | 88.3% ± 3.2 |

### Supplementary Table S2 Clinical phenotype of neural tube defects in 13 trios

| **Trios** | **Neural tube defect phenotype** | **Other phenotype** |
| --- | --- | --- |
| 1 | anencephaly & spina bifida |  |
| 2 | anencephaly & spina bifida* | low position of ear; no neck |
| 3 | anencephaly & spina bifida* |  |
| 4 | anencephaly & spina bifida | low position of ear; no neck |
| 5 | anencephaly |  |
| 6 | anencephaly & spina bifida* |  |
| 7 | anencephaly & spina bifida* |  |
| 8 | anencephaly & spina bifida* | low position of ear |
| 9 | anencephaly & spina bifida | auricular defect |
| 10 | anencephaly & spina bifida* |  |
| 11 | anencephaly & spina bifida |  |
| 12 | anencephaly |  |
| 13 | anencephaly & spina bifida* | low position of ear; no neck; cleft palate |

* craniorachischisis

### Supplementary Table S3 Clinical phenotype of neural tube defects in a cohort of 502 cases

| **Neural tube defect phenotype** | | **No. of cases** |
| --- | --- | --- |
| anencephaly |  | 209 |
|  | anencephaly & spina bifida | 79 |
|  | anencephaly & encephalocele | 1 |
|  | anencephaly | 125 |
|  | anencephaly & spina bifida &  encephalocele | 4 |
| others |  | 293 |
|  | spina bifida & encephalocele | 15 |
|  | spina bifida | 232 |
|  | encephalocele | 46 |
| total |  | 502 |

## SUPPLEMENTARY FIGURES

### Supplementary Figure S1

Bam file scripts of de novo variants identified in NTD-affected trios


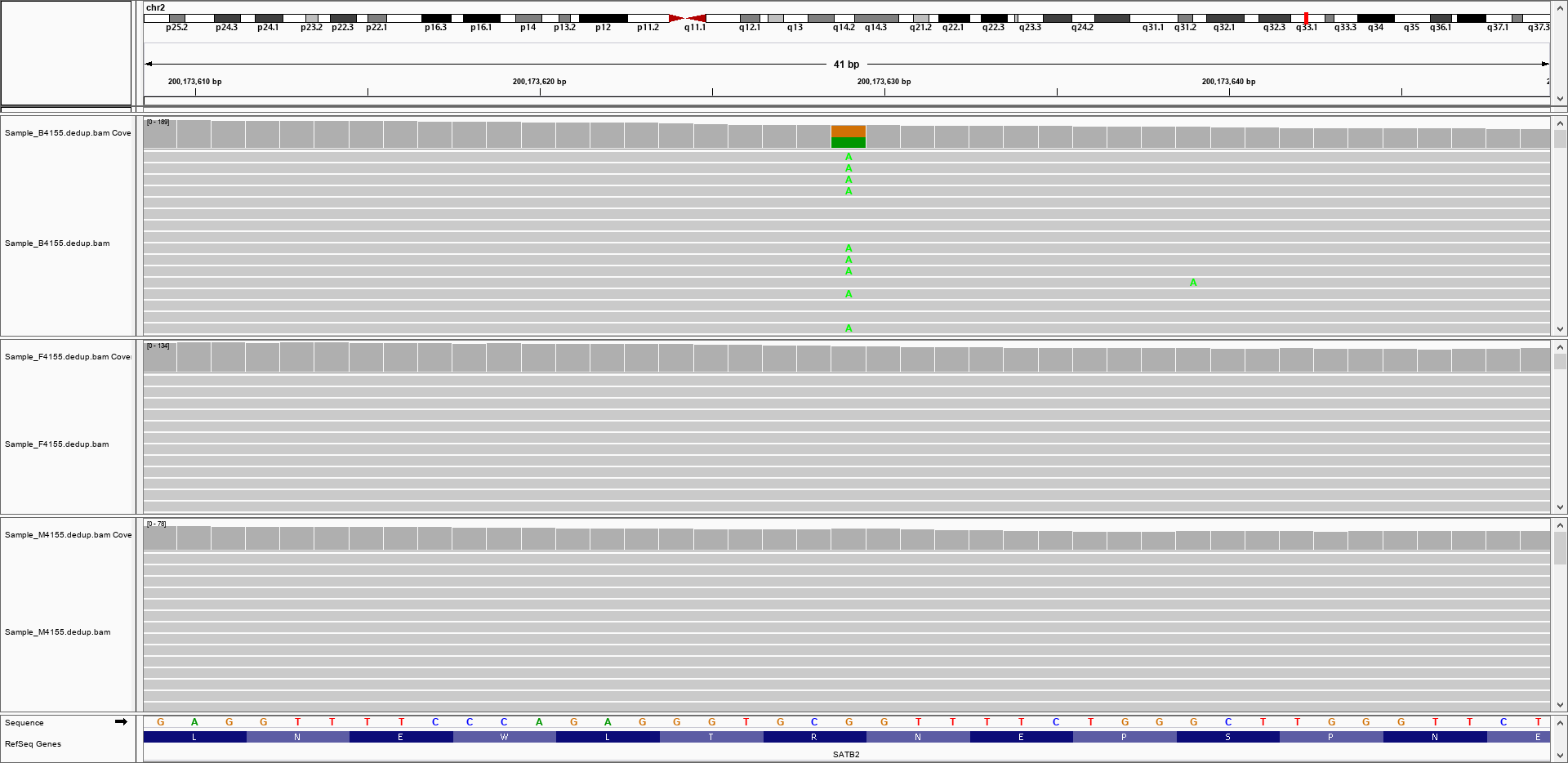


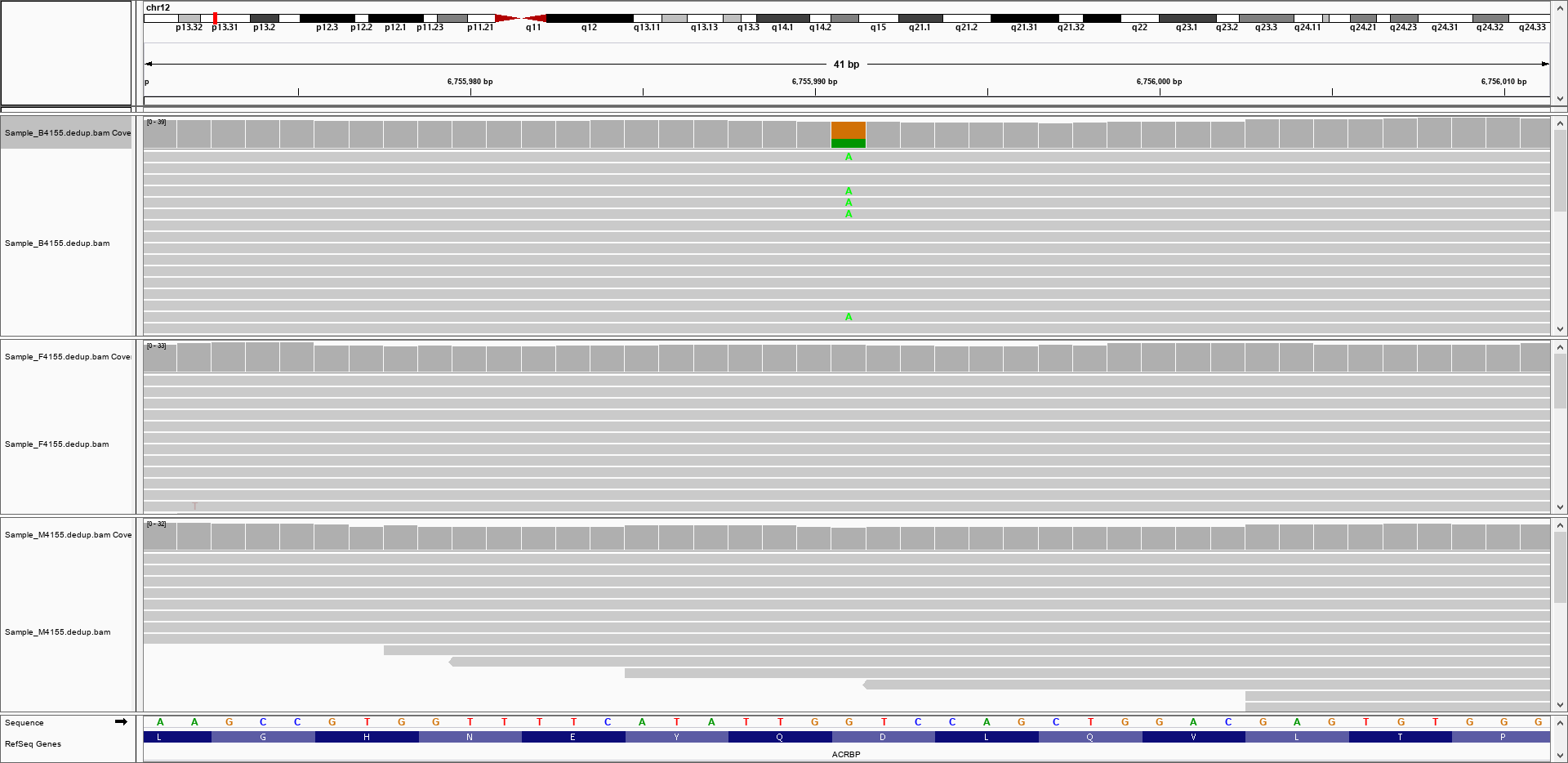


B03_ACRBP B03_SATB2


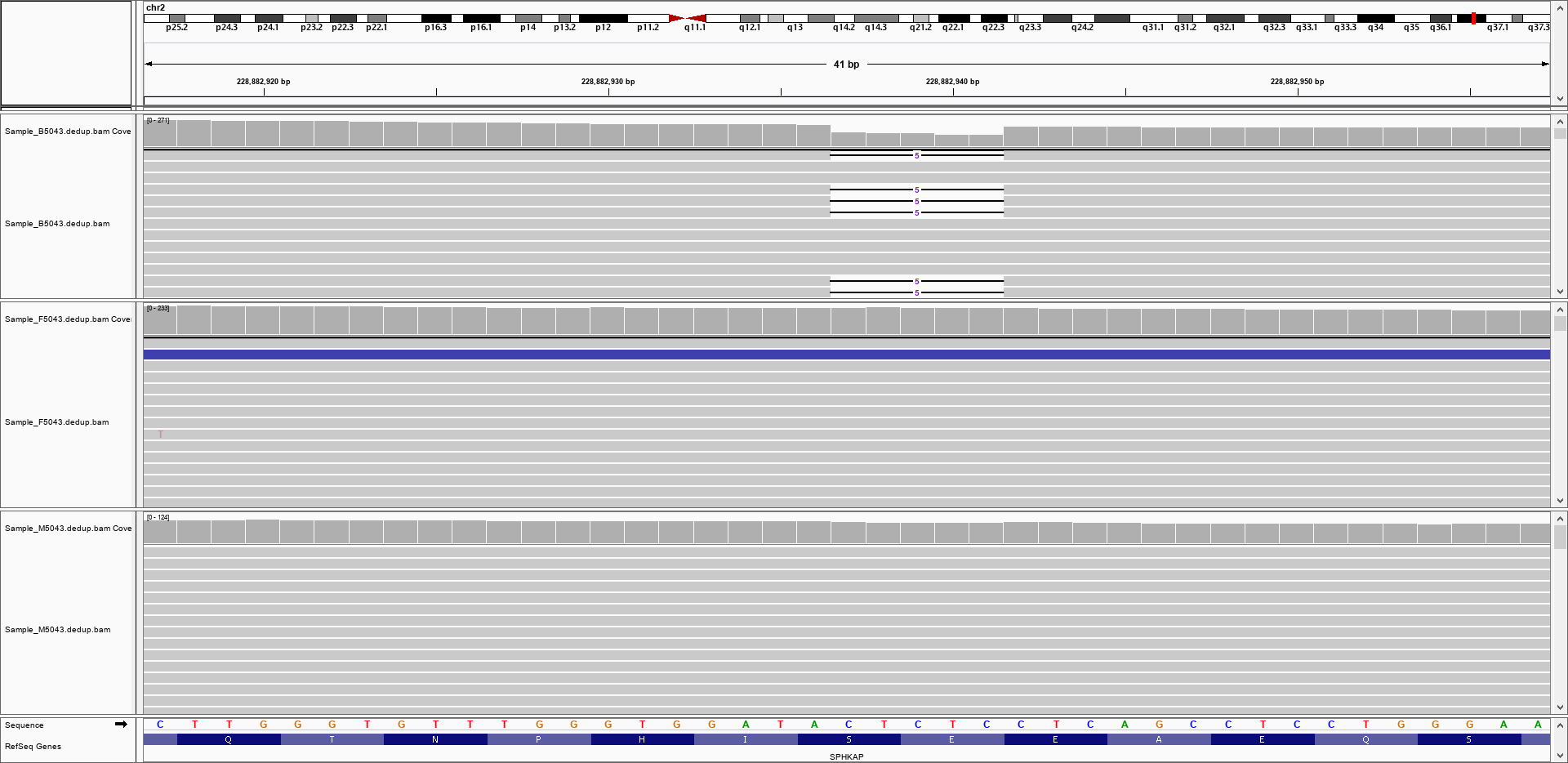

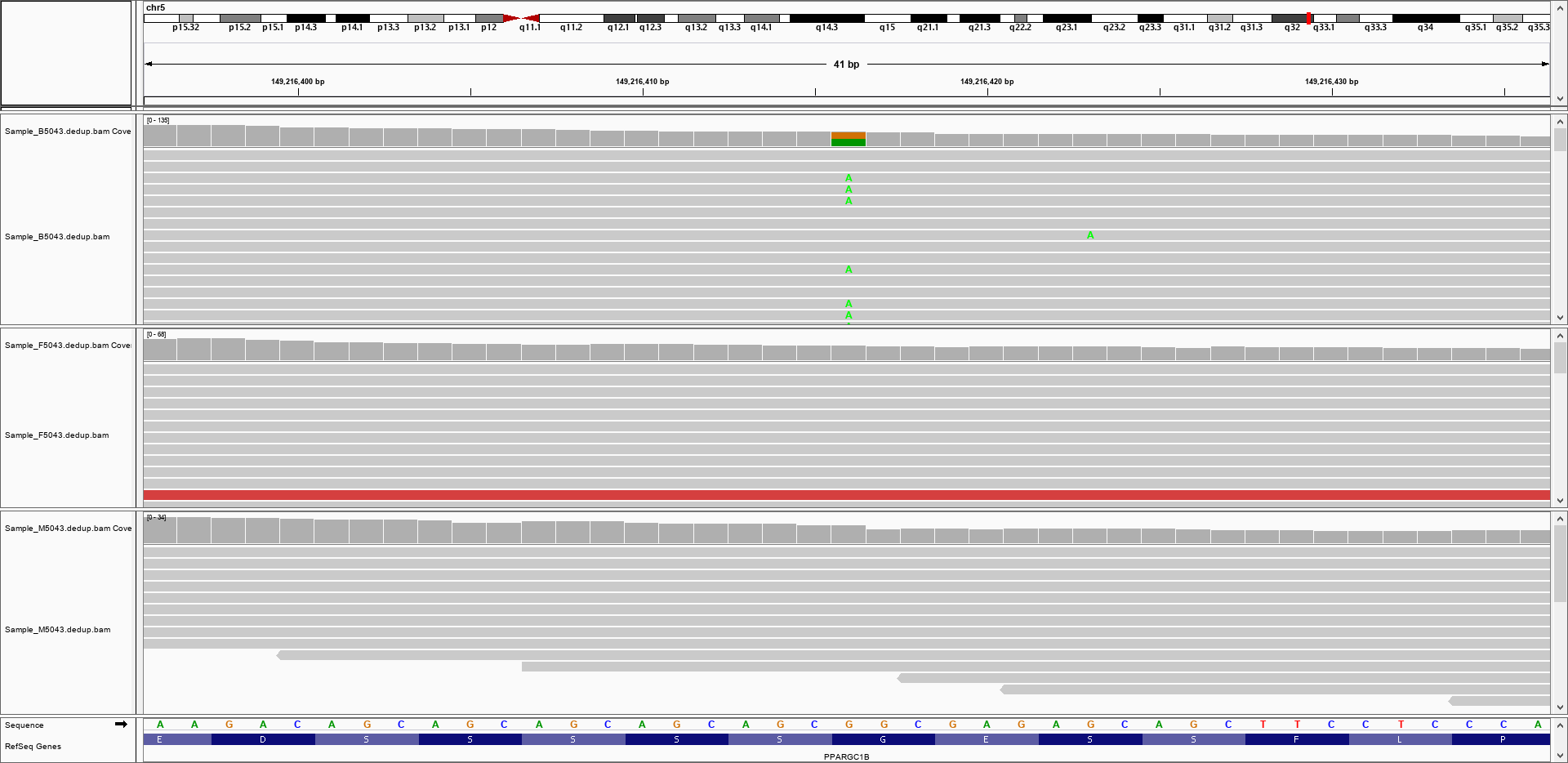

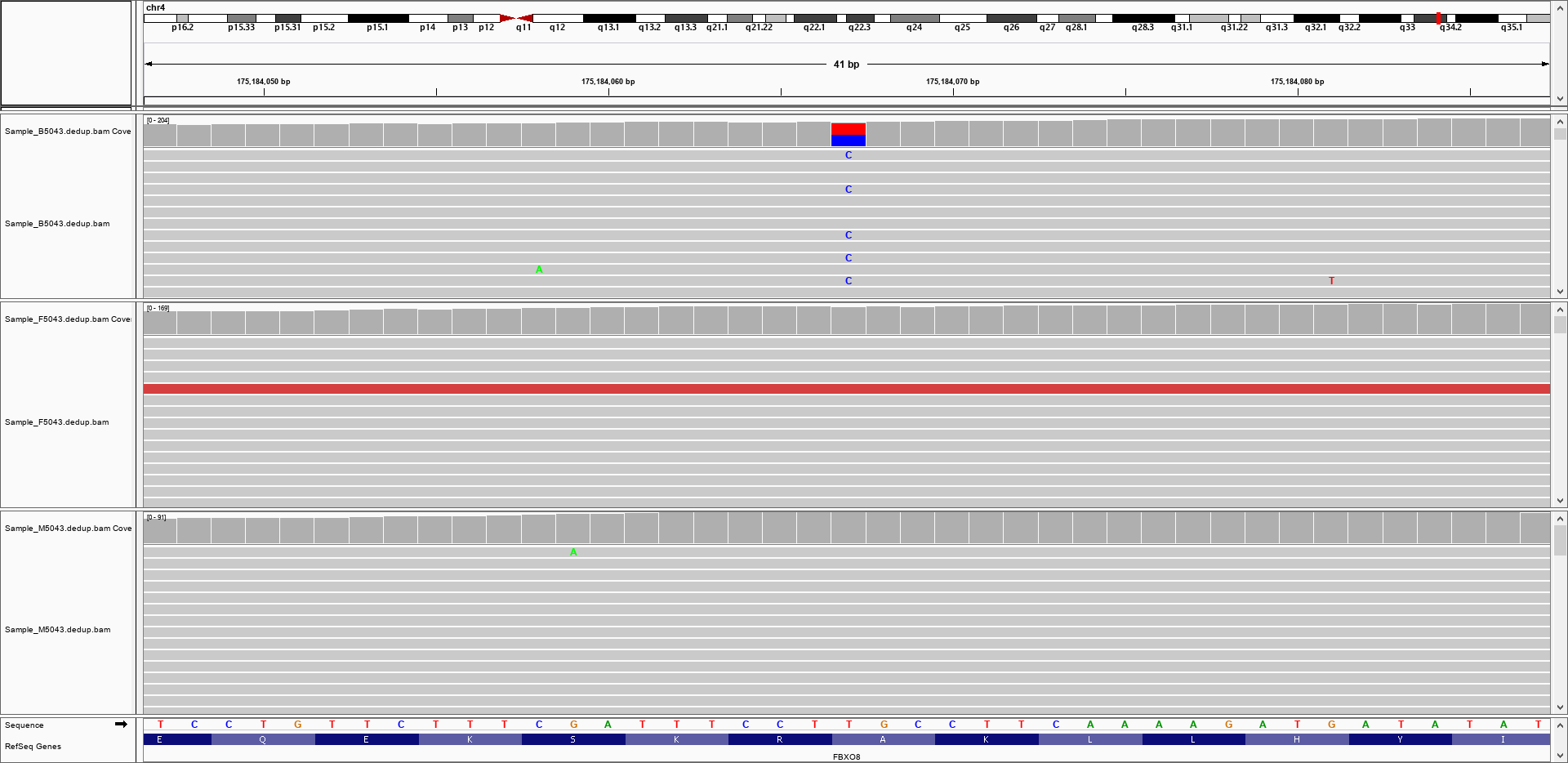

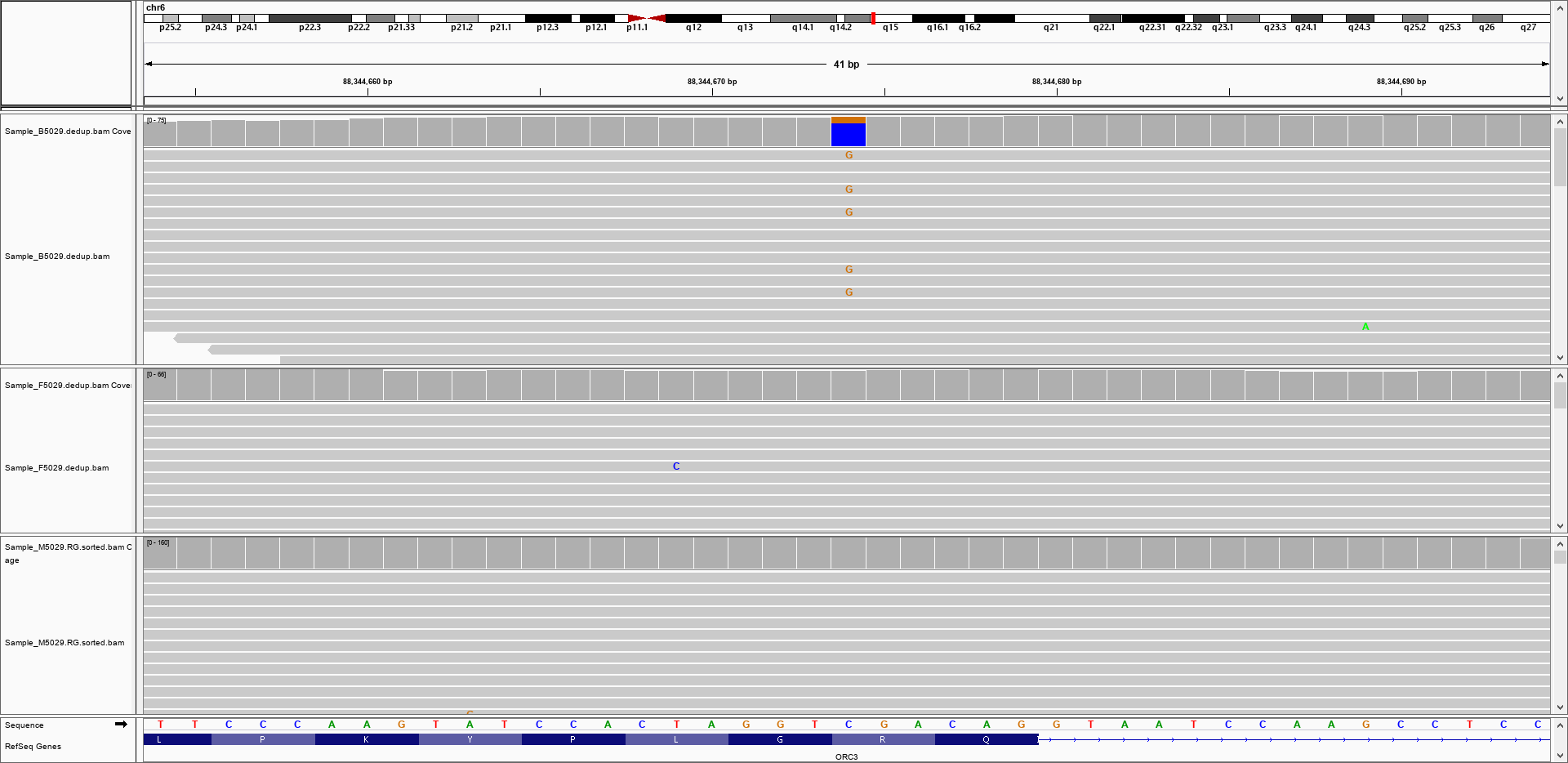
B05_ORC3 B06_FBXO8

B06_PPARGC1B B06_SPHKAP


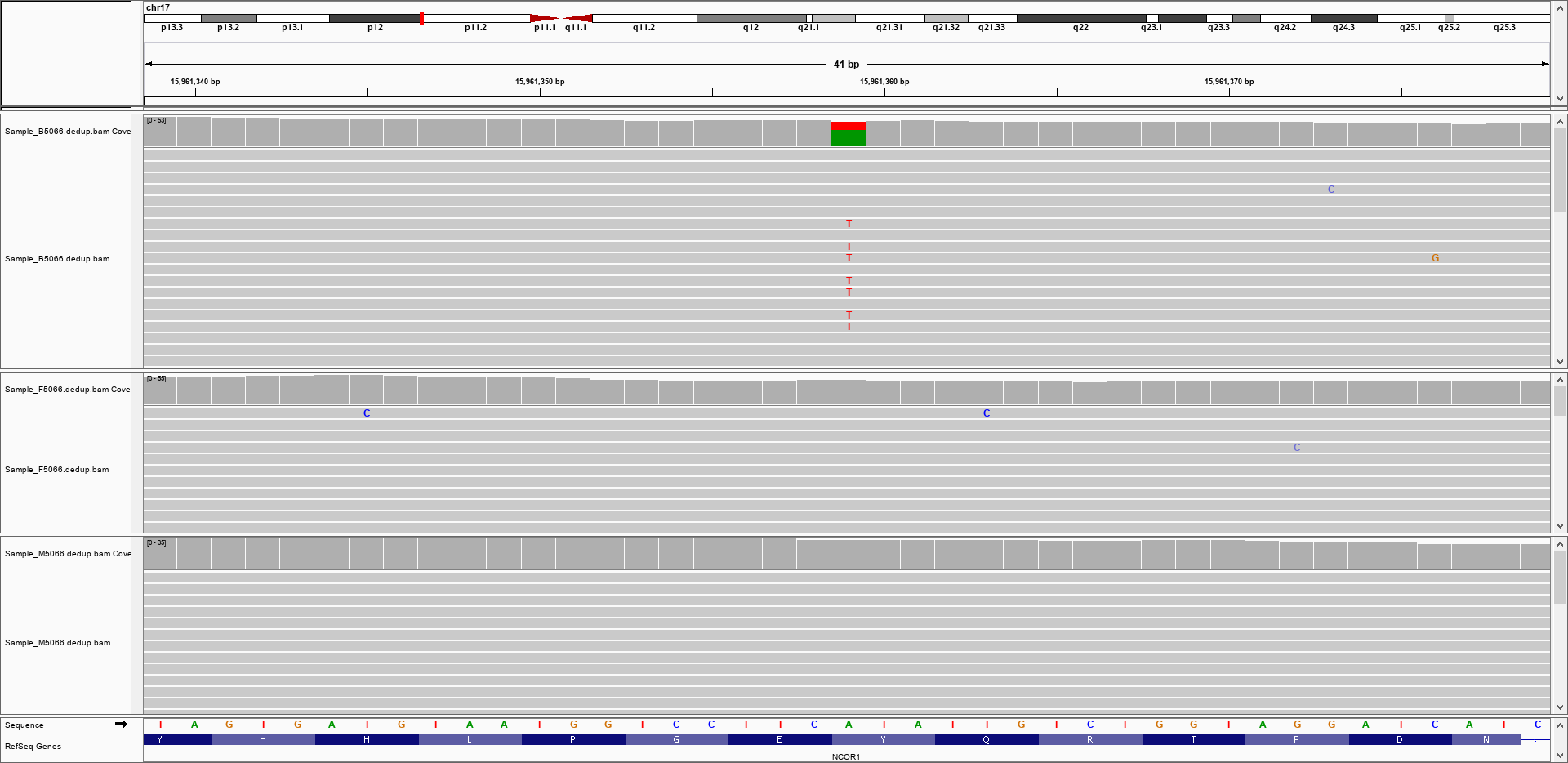

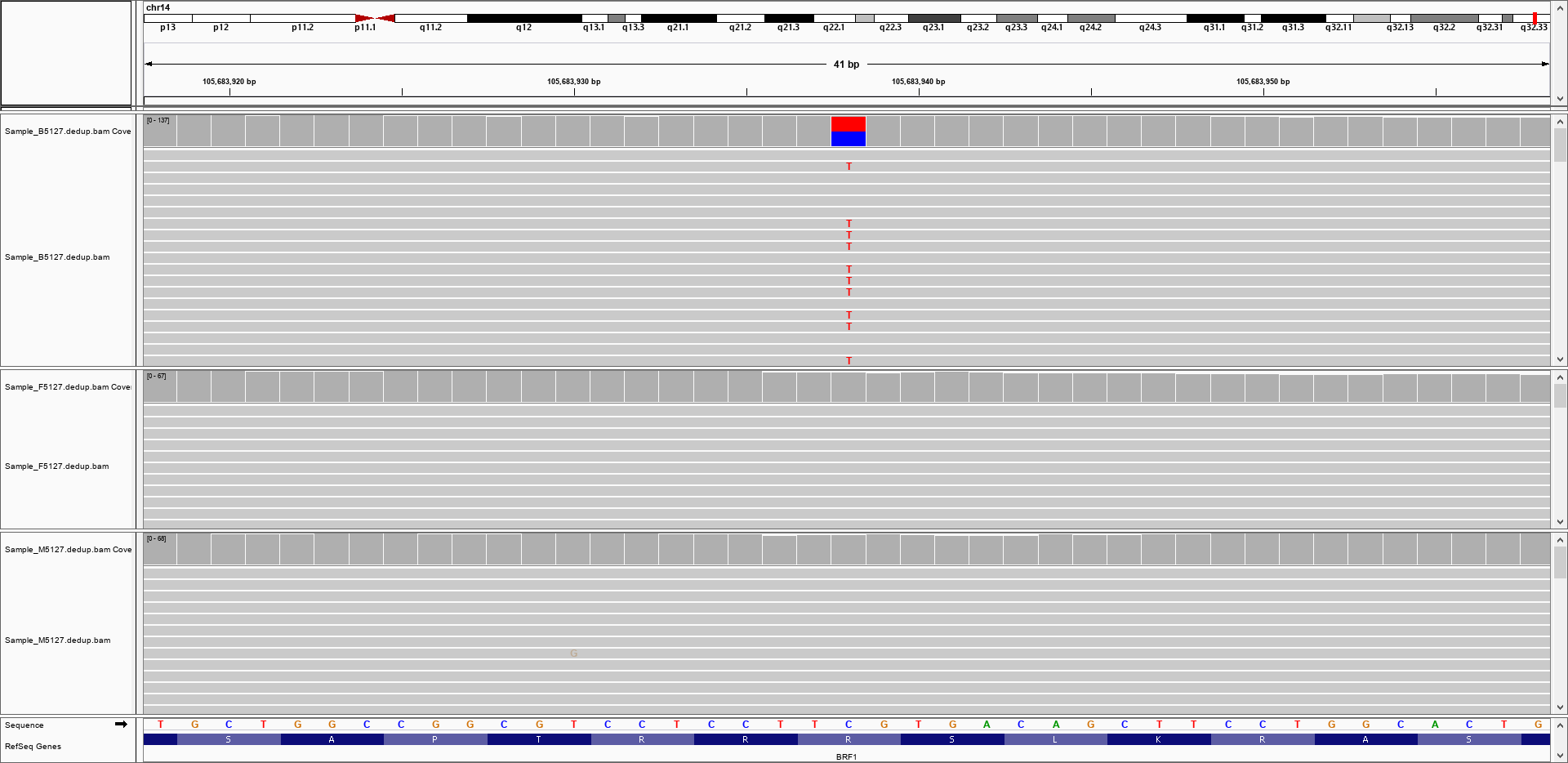

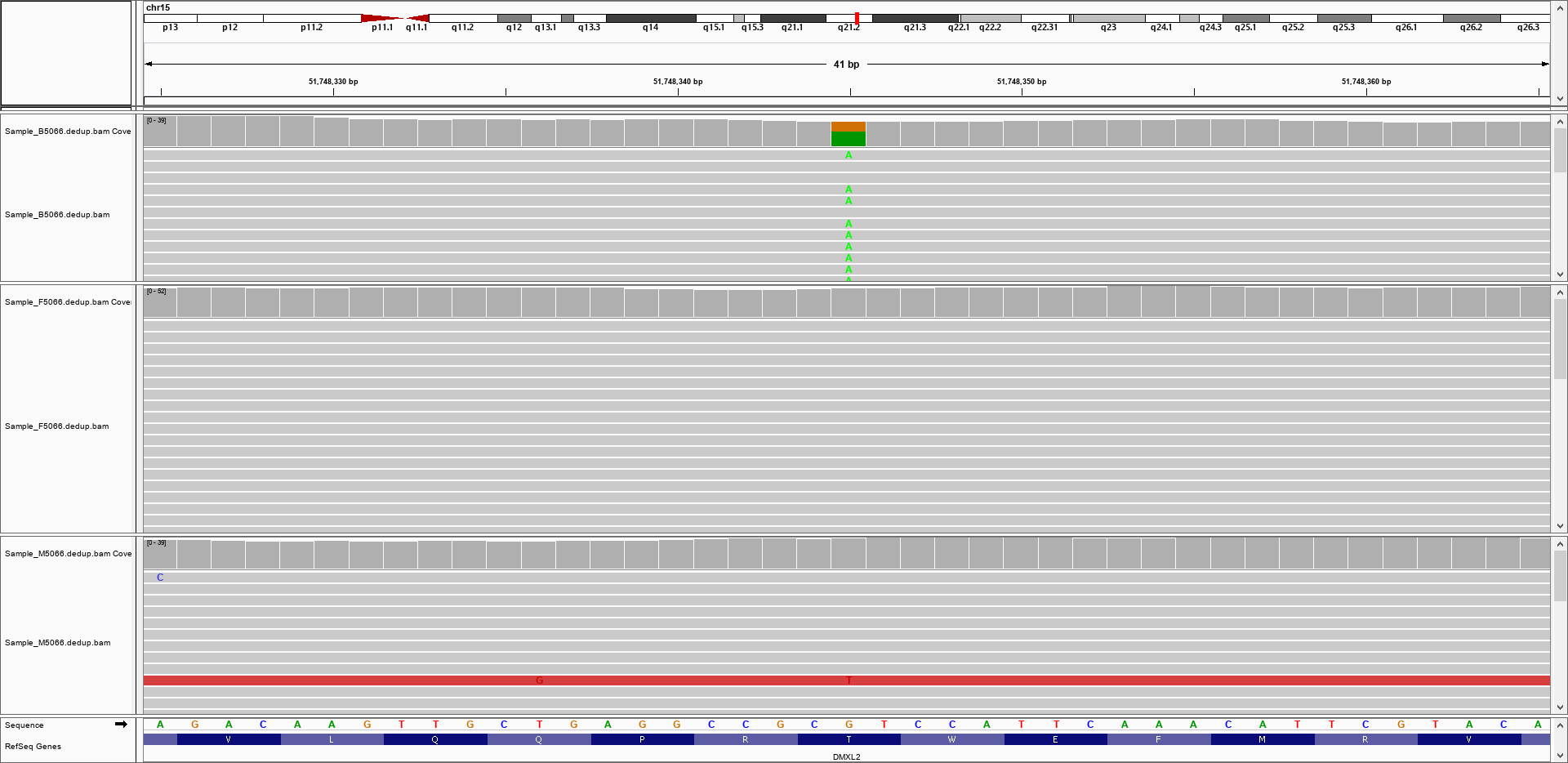

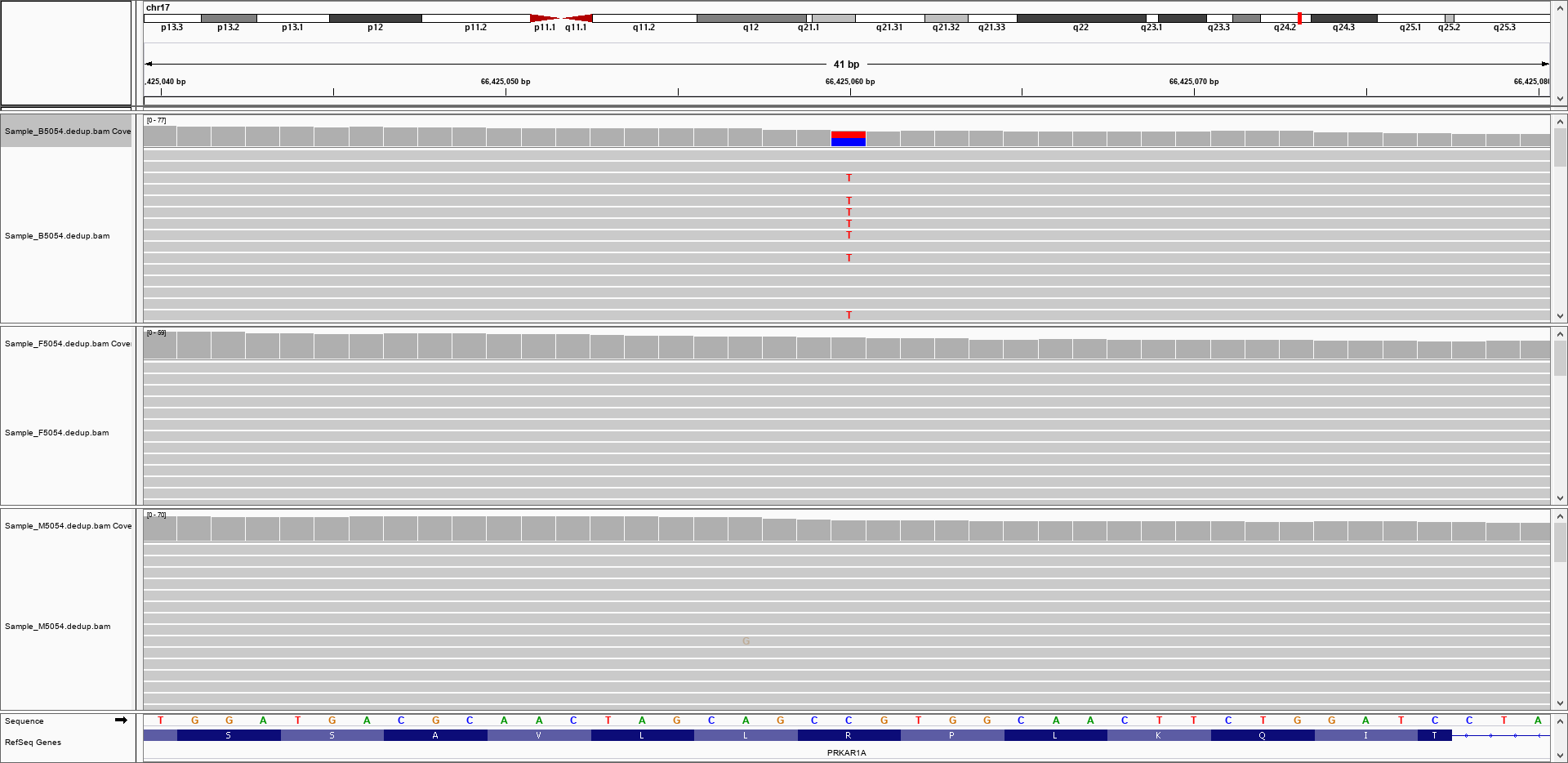
B07_WIPI1 B08_DMXL2

B08_NCOR1 B11_BRF1


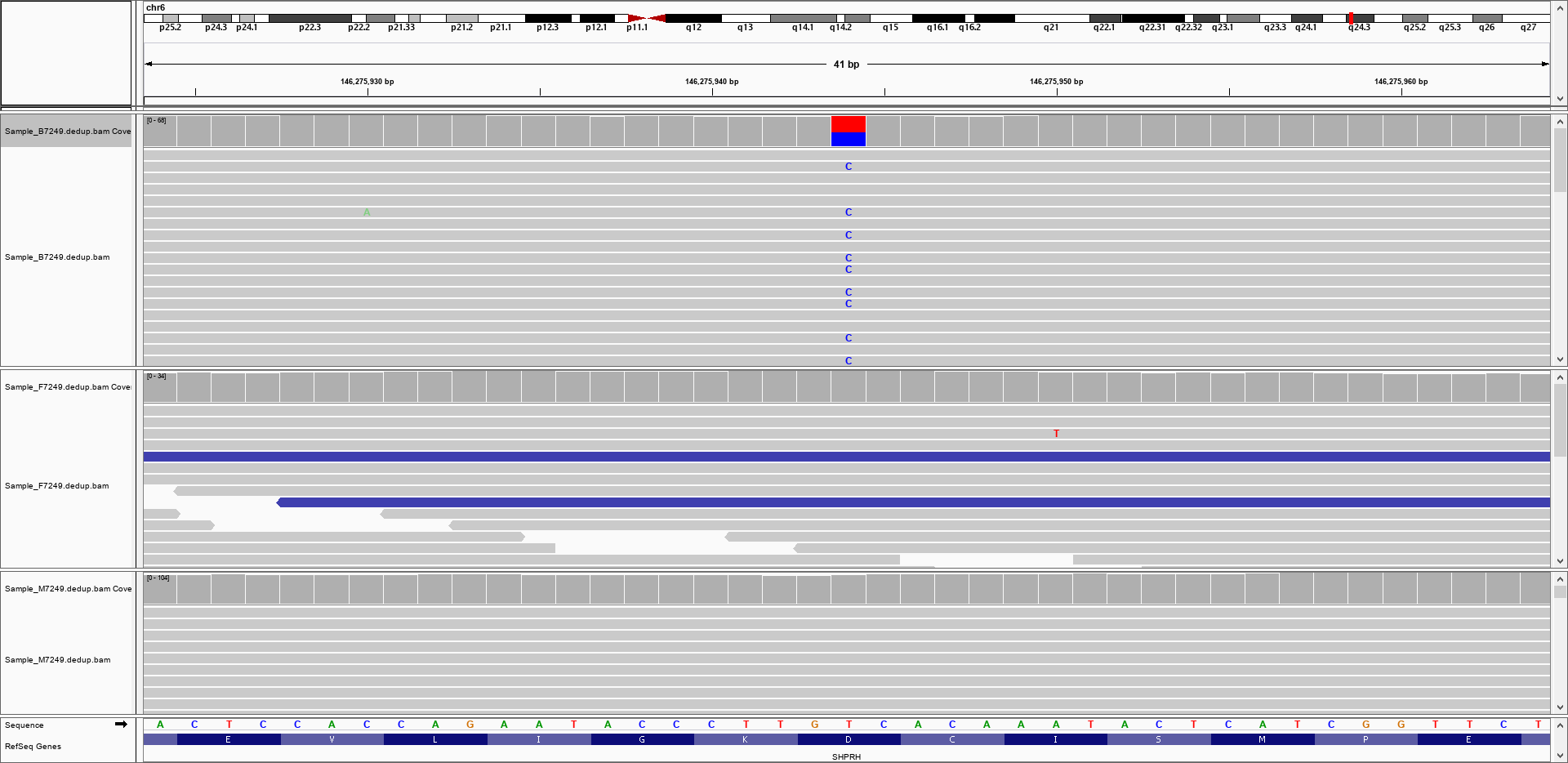

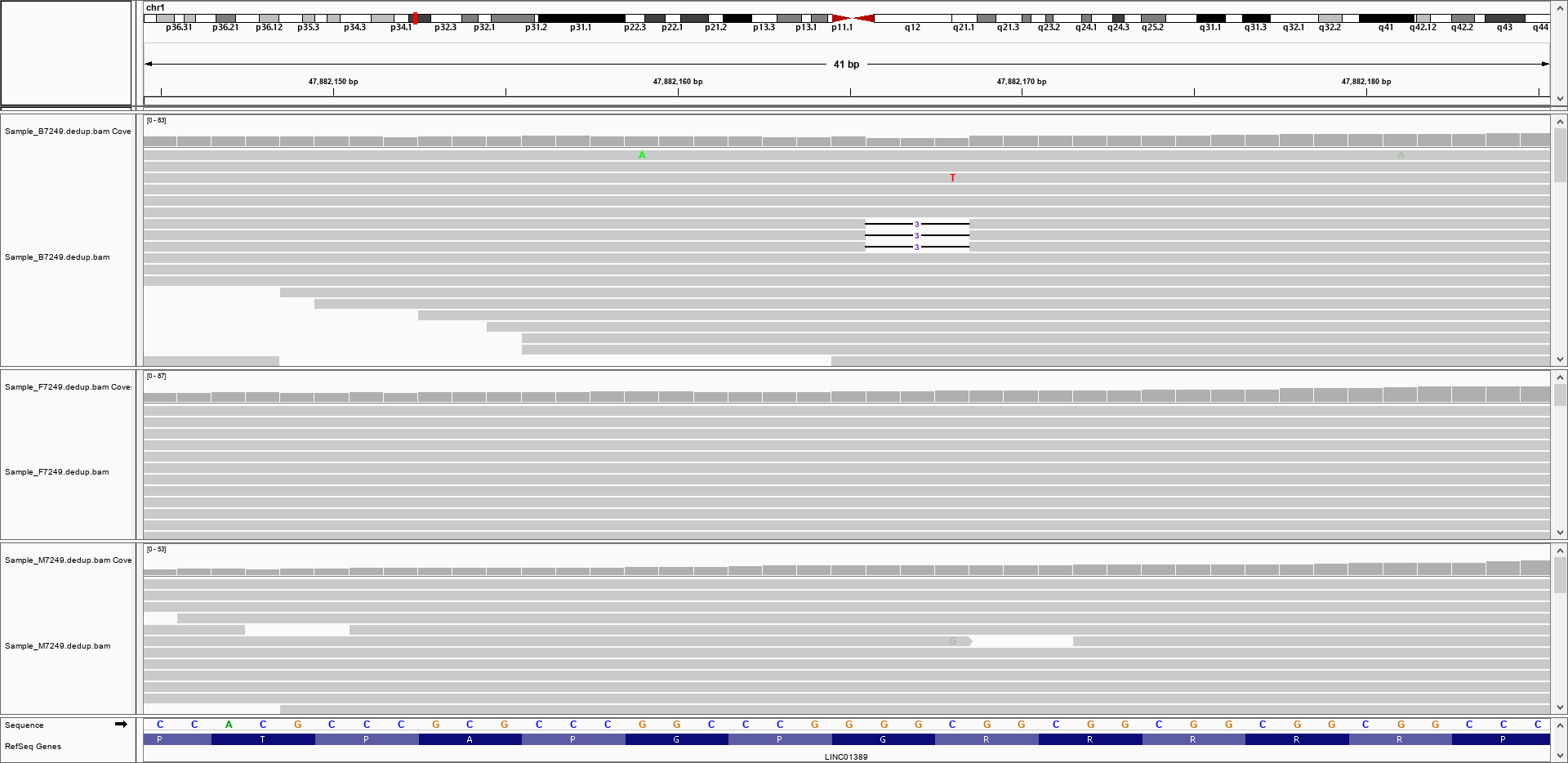

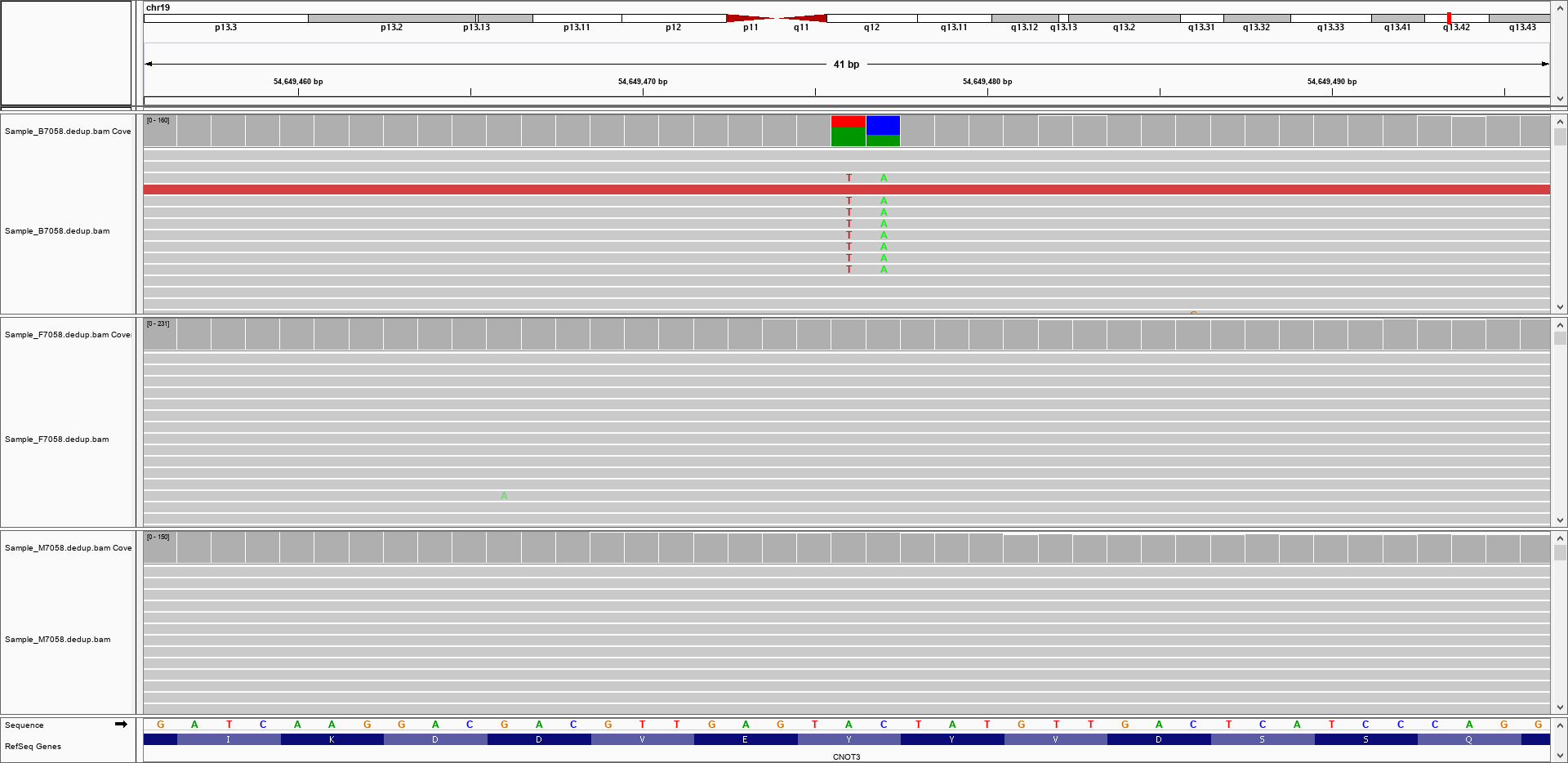
B12_CNOT3 B13_FOXE3

B13_SHPRH

### Supplementary Figure S2

Conservation analysis of WIPI1 substitution. Alignment of WIPI1 amino acid sequence between human (human NP_060453.3: p.R328Q) and other vertebrates using Clustal Omega—multiple sequence alignment, EMBL-EBI (http://www.ebi.ac.uk/Tools/msa/clustalo/). The WIPI1 variants found in NTDs affect conserved residues. National Center for Biotechnology Information accession numbers are NP_060453.3 for human WIPI1, NP_666052.1﻿ for mouse WIPI1, NP_001120769.1 for rat WIPI1, XP_001165276.1﻿﻿ for Chimpanzee WIPI1, XP_548021.3﻿ for dog WIPI1,﻿ NP_956685.1﻿ for Zebrafish WIPI1.


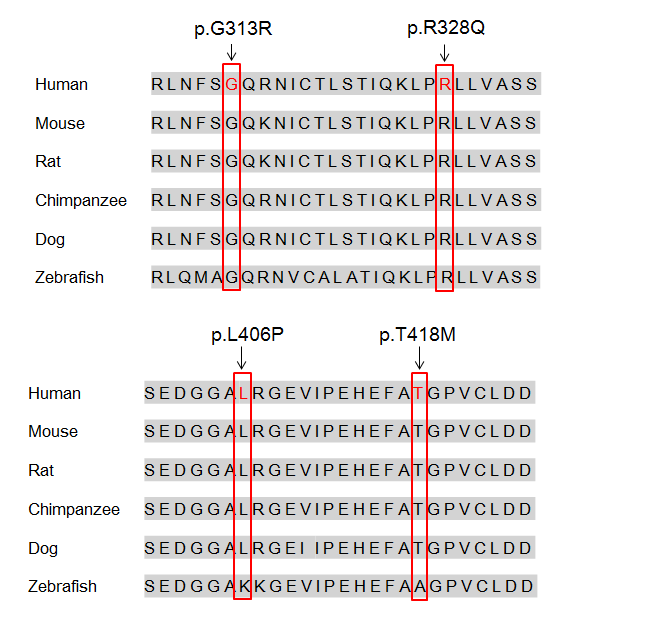


### Supplementary Figure S3

HEK293T cells were transfected with pIRES2-dsRed in combination with *WIPI1* wild-type or variants. The photographs were taken under white light (left) and fluorescence (right).


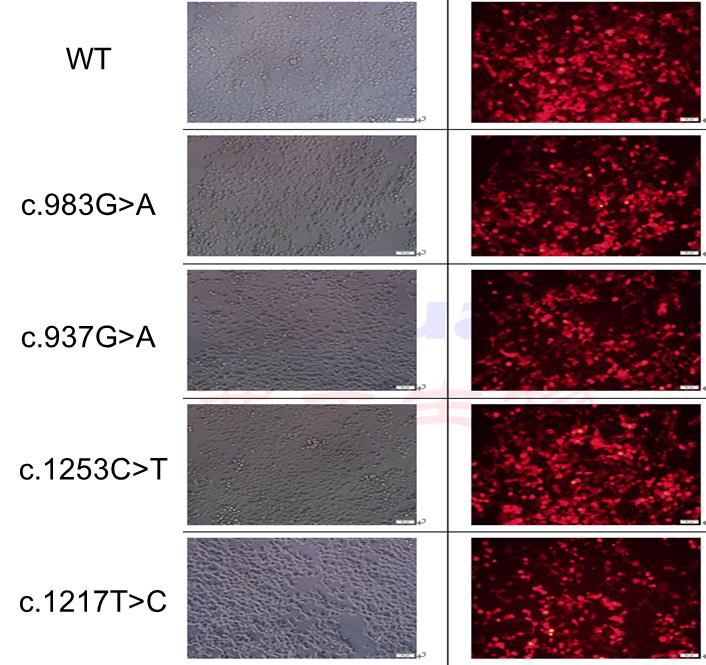


### Supplementary Figure S4

Subcellular localization of GFP- *WIPI1* wild type and variants. HEK293T cells were transfected with pIRES2-dsRed plasmids in combination with *WIPI1* wild-type or variants. Green indicated GFP-*WIPI1* and its mutant, blue indicated cell nucleus. No difference was found between wild-type and any variant in subcellular localization. Scale bar, 100 um.
